# Advancements in Lithography Techniques and Emerging Molecular Strategies for Nanostructure Fabrication

**DOI:** 10.3390/ijms26073027

**Published:** 2025-03-26

**Authors:** Prithvi Basu, Jyoti Verma, Vishnuram Abhinav, Ratneshwar Kumar Ratnesh, Yogesh Kumar Singla, Vibhor Kumar

**Affiliations:** 1 Department of Electrical Engineering, Texas A&M University, College Station, TX 77843, USA; prithvi.basu@exchange.tamu.edu (P.B.); jyotiverma607@tamu.edu (J.V.); 2 Department of Electrical Engineering, Indian Institute of Technology Bombay, Mumbai 400076, India; vishnuram.abhinav@gmail.com; 3 Department of Electronics and Communication Engineering, Meerut Institute of Engineering and Technology, Meerut 250005, India; ratnes123@gmail.com; 4 School of Engineering, Math and Technology, Navajo Technical University, Crownpoint, NM 87313, USA; ysingla@navajotech.edu

**Keywords:** lithography, nanostructure, extreme UV lithography, electron beam lithography (EBL), X-ray lithography (XRL), ion beam lithography (IBL), nanoimprint lithography (NIL), nanofabrication

## Abstract

Lithography is crucial to semiconductor manufacturing, enabling the production of smaller, more powerful electronic devices. This review explores the evolution, principles, and advancements of key lithography techniques, including extreme ultraviolet (EUV) lithography, electron beam lithography (EBL), X-ray lithography (XRL), ion beam lithography (IBL), and nanoimprint lithography (NIL). Each method is analyzed based on its working principles, resolution, resist materials, and applications. EUV lithography, with sub-10 nm resolution, is vital for extending Moore’s Law, leveraging high-NA optics and chemically amplified resists. EBL and IBL enable high-precision maskless patterning for prototyping but suffer from low throughput. XRL, using synchrotron radiation, achieves deep, high-resolution features, while NIL provides a cost-effective, high-throughput method for replicating nanostructures. Alignment marks play a key role in precise layer-to-layer registration, with innovations enhancing accuracy in advanced systems. The mask fabrication process is also examined, highlighting materials like molybdenum silicide for EUV and defect mitigation strategies such as automated inspection and repair. Despite challenges in resolution, defect control, and material innovation, lithography remains indispensable in semiconductor scaling, supporting applications in integrated circuits, photonics, and MEMS/NEMS devices. Various molecular strategies, mechanisms, and molecular dynamic simulations to overcome the fundamental lithographic limits are also highlighted in detail. This review offers insights into lithography’s present and future, aiding researchers in nanoscale manufacturing advancements.

## 1. Introduction

Semiconductor chip manufacturing involves various techniques and processes performed on a substrate. Lithography is a crucial step accounting for nearly one-third of the total manufacturing cost. As chip density increases, device sizes must shrink to keep pace with Moore’s Law. Achieving this miniaturization requires an optimal lithography method with the necessary resolution [1]. A lithography system comprises an optical system, a mask, resists, and an exposure setup. The optical system includes a lens array, a light source, and sensors [2]. Masks, typically made from glass or quartz, are coated with chromium or iron oxide. Patterns are generated on the mask using maskless writing technologies and then transferred to the resist on the wafer [3,4].

Resists are complex organic compounds that undergo chemical changes when exposed to light or photons. Photoresists are classified as positive or negative tone. In positive tone resists, the exposed regions are washed away during development [5]. This produces patterns identical to those on the mask. In contrast, negative tone resists undergo crosslinking, reducing solubility in the exposed regions and creating patterns that are the inverse of the mask [6,7]. Due to their superior resolution, positive tone resists are widely used in IC fabrication. Optical lithography historically employs UV lamps with wavelengths of 365 nm and 436 nm for exposure [8].

To construct nanometer-sized devices for cutting-edge semiconductor integrated circuits (ICs) and almost any other fundamental application across multiple scientific disciplines, the production or imprint of nano-sized patterns on a substrate, known as nanolithography, is essential [9,10]. This technique is not only a fundamental driving technology in semiconductor and integrated circuit manufacturing, but it also plays an important role in the fabrication of commercially available microelectromechanical system (MEMS) and nanoelectromechanical system (NEMS) devices, which are used in automobiles, optical switches, microphones, mobile phone chips, and biosensors. Nanolithography has been used to successfully create semiconductor devices such as MOSFET, CMOS, and NMOS, as well as memory devices such as DRAM and MRAM [11,12]. For the reasons stated above, excimer lasers were introduced, which used DUV wavelengths of 248 and 193 nm [13]. However, the resolution was limited by diffraction effects; therefore, more advanced techniques such as short-wavelength EUV lithography, electron beam lithography, X-ray lithography, ion beam lithography, and nanoimprint lithography gained popularity.

One of the methods to fabricate complex features with variable dimensions ranging from a few microns to a sub-100 nm scale is the Mix and Match (M&M) approach [14]. This technique involves combining two or more patterning techniques to improve throughput. The Mix and Match (M&M) approach is a potential technique to manufacture devices in advanced research areas like quantum technologies and photonic integrated circuits (PICs) [15,16]. Other direct-writing technologies like two-photon polymerization (2PP) and laser direct writing are capable of fabricating high-resolution 3D structures [17]. 2PP is a non-linear technique used in 3D printing and microfabrication, where polymer materials are selectively crosslinked using laser light. 2PP has been implemented to fabricate structured biocompatible 3D nanogrid structures to enhance neuronal directional growth [18,19]. The versatility of the 2PP technique makes it an excellent choice for advanced applications in fields such as microelectronics, biotechnology, and optics [20].

Laser direct write lithography using near-i-line wavelength is a high-throughput and cost-effective maskless technique for microscale fabrication, directed towards low-volume production. Laser direct writing applications including processors, energy harvesting and storage, sensing, and bioelectronics have been explored [21,22]. Soft lithography, a cost-effective, flexible process, consists of a wide variety of techniques based on printing, molding, and embossing with an elastomeric stamp [23]. Wet lithography is another technique that is capable of patterning large areas of microstructures of organic and inorganic soluble materials and biological compounds in buffer solutions. This technique does not require any specialized facilities or tools and can be achieved using compact discs and microscopy grids [24]. The discovery of Scanning Probe Microscopy (SPM) also boosted the advancements of nanotechnology in mapping the surface topography and studying the physical and chemical properties of sub-nanometer molecular structures [25].

The first manufacturable multi-beam system for scanning electron microscopy was invented in 2005, featuring 100 beams of sub-10 nm size. Around the same time, IMS Nanofabrication (Wien, Austria) introduced its own multi-beam concept for projection mask-less lithography and later for photomask patterning [26]. IMS’s electron multi-beam system was an evolution of its earlier ion multi-beam technology. By 2015, IMS’s electron multi-beam program achieved its goals, demonstrating the ability to pattern a full 6-inch mask in high-volume manufacturing, marking a significant shift in photomask patterning technology [26,27]. This innovation arrived just in time to complement the deployment of EUV lithography, overcoming limitations of resolution and write time seen with Variable Shaped Beam (VSB) technology [28,29].

In 2016, IMS launched the MBMW-101, the first high-volume manufacturing (HVM) version, marking a breakthrough in mask writing technology. This was followed by the introduction of the MBMW-201 in 2019, a second-generation tool that further enhanced throughput and resolution [30]. The MBMW-201 has been used for EUV mask production at 7 nm, 5 nm, and 3 nm nodes, as well as research for the 2 nm node [31]. NuFlare Technology (NFT) has developed several mask writers, including the MBM-2000 and MBM-2000PLUS, which are used for producing advanced optical and EUV masks. In 2023, NFT launched the MBM-3000, designed for the N2 technology node [32]. More recently, researchers have developed a mask process correction (MPC) system integrated into the multi-beam writer. This advancement, coupled with the introduction of inverse lithography technology (ILT), has ushered in a new era of photomask design, transitioning mask patterns from rectilinear to curvilinear shapes [33].

In recent decades, much research and development has been conducted to advance nano-lithographic processes. Even though optical lithography has accounted for more than 40% of lithographic operations in the last five years, alternative nano-lithographic techniques have achieved substantial advances in terms of resolution capabilities, new and superior resists, and developing materials [34]. In the current review, a variety of modern lithography techniques like extreme ultraviolet lithography, electron beam lithography, X-ray lithography, ion beam lithography, and nanoimprint lithography are discussed along with the role of alignment marks in ensuring precise layer-to-layer registration, emphasizing the innovations and various techniques suitable for lithography systems. The mask fabrication process is also touched upon, detailing the materials, types, innovations in various mask materials, and challenges in producing defect-free masks with emphasis on advancements in their achievable resolution in recent years. Finally, the emerging molecular strategies including the molecular structure and properties of resist materials, the advanced photochemical reactions involved in the photoresist chemistry, and predicting the material behavior at nanoscale via molecular simulations are also described in detail.

## 2. Extreme Ultraviolet Lithography

Extreme ultraviolet lithography (EUVL), with a wavelength of approximately 13.5 nm, was introduced in 1988 and has been widely employed in the semiconductor industry since 2018. Because of the Rayleigh diffraction effect, smaller wavelengths of light have been used to achieve a reduced feature size with excellent resolution via a lens system, resulting in a smaller semiconductor device. Multi-patterning technologies enable IC production to extend beyond 3 nm [35]. Nguyen et al. [36] fabricated NMOS transistors with minimal gate length variations of 0.075, 0.1, 0.11, 0.12, and up to 0.18 µm to quantitatively assess the lithographic efficacy of the EUVL laboratory apparatus. This experiment utilized three distinct gate oxide thicknesses: 25 Å, 40 Å, and 55 Å. Each device field comprised 24 devices, 23 transistors, and a 50 × 50 µm^2^ capacitor, with a maximum transistor gate length of 20 µm.

**Figure 1:**
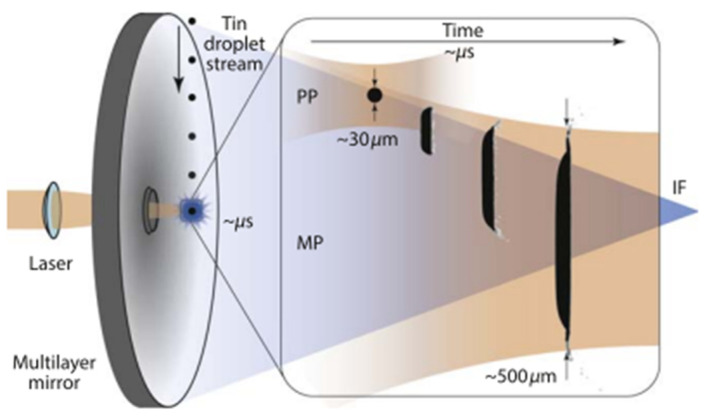
Schematic diagram of a laser–droplet interaction in an industrial EUV light source module. Copyright 2019 IOP Publishing. Reproduced with permission from [38]. All rights reserved.

An EUV system comprises a source, a patterning mask, an exposure apparatus, and multilayer mirrors. The EUV source primarily comprises discharge-produced plasma (DPP) sources or laser-produced plasma (LPP) sources capable of generating electron temperatures over 100,000 K. The EUV source, utilizing laser-produced plasma (LPP) technology, comprises multiple components, including a carbon dioxide (CO_2_) laser. Research demonstrated that tin plasma EUV can be stimulated by a CO_2_ laser pulse at a wavelength of 10.6 µm due to its superior conversion efficiency, reduced debris generation, and elevated average power levels, all while mitigating significant beam distortion issues typically associated with solid-state lasers at high intensities. Elevated conversion efficiency is achieved through the use of brief laser pulses and increased repetition rates. The conversion efficiency of the EUV source is dependent on the effectiveness of plasma formation and heating [37]. The schematics illustrating the laser-droplet interaction in an industrial EUV light source module are depicted in Figure 1 [38].

Reflective mirrors, rather than refractive lenses, are utilized for the optical components that facilitate the imaging functions of the scanner. These mirrors feature surface coatings comprising up to 100 alternating layers of silicon and molybdenum, which reflect light through interlayer interference. Notably, these coatings demonstrate exceptional performance with light at a wavelength of 13.5 nm. However, each mirror is theoretically limited to reflecting only 72% of the incident EUV light, with the remainder being absorbed by the mirror [39].

To selectively filter 13.5 nm EUV from the plasma and enhance reflective rates, the collector surface is coated with several Mo/Si layers. The reflection wavelength λ can be accurately determined by adjusting layer thickness and reflection angle. For a wavelength of 13.5 nm, approximately 40 pairs of multilayer mirrors are employed, featuring a thickness of about 270 nm, an angle of incidence of 6°, and a bilayer spacing close to 7 nm. A standard multilayer mirror for the EYVL system consists of around 40 pairs of Mo/Si layers.

Various multilayer mirrors, including Ru/B_4_C, Ru/C, Pd/B_4_C, V/Sc, Cr/V, and Pd/Y multilayer mirrors, have been produced by a research group at Tongji University using direct current magnetron sputtering. The theoretical reflectance for a 40-pair Mo/Si multilayer at a wavelength of 13.36 nm is calculated to be 74.47% [40]. A capping layer positioned above the Mo/Si multilayer is implemented to enhance the endurance and reliability of the EUV collector. Ruthenium has been identified as an excellent material choice for this capping layer due to its stability in EUV and hydrogen environments. Additionally, a research group at ASML has explored other advanced materials that exhibit strong EUV transmission and thermal stability, concluding that polysilicon and metal silicide films perform well in high-temperature conditions [41].

ASML has recently devised an innovative technique for merging many small droplets into larger ones at increased separation distances. ASML has launched its third-generation droplet generator, including a droplet diameter of 27 μm and an average lifespan of around 2700 h [42]. ASML is presently examining the next phase of laser power enhancement. Recent advancements have led to the achievement of a dose-controlled EUV power output of 420 W at a high duty cycle. In addition, ASML’s next-generation NXE:3600D has reached an open-loop EUV power level of 530 W, while the degradation of the EUV collector has been observed to remain below 0.05% [43].

The latest high-power CO_2_ laser, with master oscillator power amplifier (MOPA) pre-pulse technology, is considered the optimal solution, capable of producing a laser power output exceeding 40 kW. MOPA pre-pulse technology can deliver a stabilized EUV power of 250 W, achieving a conversion efficiency of 6% and maintaining a stable dose with an error margin of less than 0.1%. Academic and industrial research and development teams are actively exploring innovative approaches for the advancement of EUV sources. One promising strategy involves the integration of a multifiber laser coherent synthesis system with a nano-Sn generator. This combination is expected to provide significant benefits in achieving high peak power, high average power, and enhanced conversion efficiency simultaneously [44].

### 2.1. EUV Lithography Resolution

The advent of EUV scanners, now reinforcing their role as benchmark high-volume manufacturing (HVM) system, enables the industry to surpass the 10 nm resolution barrier in a single exposure. To achieve the theoretical minimum limit of 0.25 for the imaging factor k_1_, the Abbe–Lin equation can be utilized to calculate the critical dimension (CD) value as follows:

CD = k_1_ × (λ/NA) = 0.25 × (13.5/0.33) = 10.3 nm

The requirement of an increased numerical aperture (NA) of the optics is essential to exceed the 10 nm resolution limit while adhering to the parameters of the EUV lithographic framework (wavelength λ = 13.5 nm). The implementation of high-NA optics, functioning at NA = 0.55, as opposed to the prior NA = 0.33, enables the production of 8 nm features while utilizing a k_1_ imaging factor of 0.325 [45]. The diminishment of the minimal feature size that may be printed, hence facilitating the ongoing contraction as prescribed by Moore’s Law, represents but one perspective on the advantages of high-NA EUV lithography [46]. The enhancement of the numerical aperture in the imaging optics has enabled the production of features that exhibit greater lithographic contrast, as measured by the normalized image log-slope (NILS). Enhancing the NILS is as crucial as the reduction, as indicated by the relationship LCDU = k_4_ × (1/NILS) × (hv/dose)^1/2^ [45], where lithographic contrast is associated with local CD uniformity (LCDU), a metric of CD variability. To achieve a sufficiently high yield with minimal defectivity, it is imperative that LCDU remains low [45,47].

### 2.2. EUV Lithography Resists

Two primary categories of resists are utilized in EUV lithography: chemically amplified resists (CARs) and non-chemically amplified resists. The main challenges facing next-generation lithography resists include resolution, sensitivity, and line edge roughness (LER). Additionally, factors such as EUV absorption, defect density, and outgassing significantly influence the efficiency of the system. Chemically amplified resists are commonly employed in 248 nm and 193 nm optical lithography, typically comprising a polymeric or molecular matrix, photoacid generators (PAGs), and base quenchers. Upon exposure to irradiation, PAGs produce acids that alter the dissolution rate of the matrix during the post-exposure bake (PEB) stage. During this process, the photogenerated acids catalyze reactions within specific groups of the matrix, leading to changes in hydrophilicity, crosslinking, or backbone scission, which ultimately affects solubility, particularly in aqueous base developers [48].

The energy of a photon at 248 nm is 5 eV, while at 193 nm it is 6 eV; in contrast, a photon at 13 nm has an energy of 93 eV. This increased energy per photon contributes to shot noise and LER. A single-layer ultrathin resist applied over a hard mask can help control line edge roughness (LER). Research indicates that reducing PAG size and increasing PAG concentration can effectively lower LER [49].

**Figure 2:**
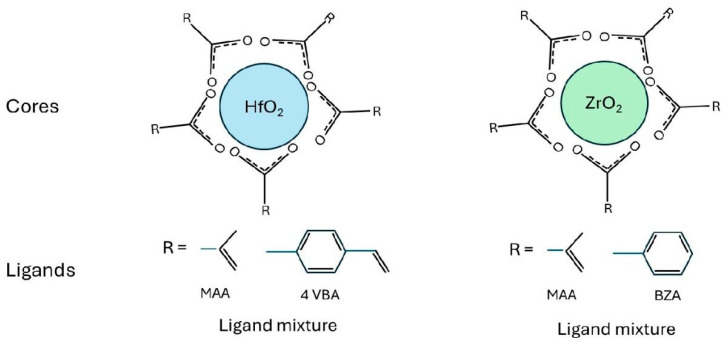
Schematic diagrams of ZrO_2_ and HfO_2_ nanoparticle with their core metal oxides and the organic ligands surrounding the cores.

On the other hand, metal oxide nanoparticles (NPs) have been proposed as next-generation photoresist materials for several key reasons related to their unique properties, which make them ideal candidates for advanced lithographic processes [50]. The main factors driving their potential are high photochemical stability, increased resolution and patterning ability, improved sensitivity and process control, reduced toxicity and environmental impact, and their compatibility with advanced lithographic techniques (e.g., 3 nm, 2 nm) [51]. The production of these nanoparticles involves carefully controlled hydrolysis of zirconium or hafnium alkoxides in an excess of carboxylic acid, followed by precipitation processes to yield ZrO_2_-NP or HfO_2_-NP with organic ligands. The NP size is kept below 3 nm, making it suitable for sub-20 nm lithography. These nanoparticles can generate both positive and negative tone patterns using either a photo radical initiator or PAG. They demonstrate significant etch resistance along with thermal and chemical stability [52]. A schematic diagram illustrating the ZrO_2_ and HfO_2_ nanoparticles, including their core metal oxides and surrounding organic ligands, is shown in Figure 2.

The nanoparticle films utilizing 2,2-dimethoxy-2-phenyl acetophenone (DPAP) as a photo-initiator have been identified as capable of functioning as both positive and negative tone photoresists. Exposure to deep-UV light (254 nm or 193 nm) followed by development in isopropyl alcohol or t-amyl alcohol produces a negative tone image. In contrast, exposure followed by post-exposure baking (PEB) at 130 °C for 3 min, followed by development in tetramethylammonium hydroxide (TMAH), results in positive tone patterns [53].

In 2010, researchers at Cornell University introduced one of the earliest metal-oxide-based hybrid resists. This photoresist features an inorganic HfO_2_ core surrounded by a shell of electro-reactive organic ligands. These resists exhibit dual-tone behavior, functioning as either positive or negative photoresists depending on the development conditions. The resist’s performance in both positive and negative tones was evaluated under identical experimental conditions with exposure to a 254 nm wavelength, achieving resolutions of 0.9 μm for positive tones and 0.8 μm for negative tones.

**Figure 3:**
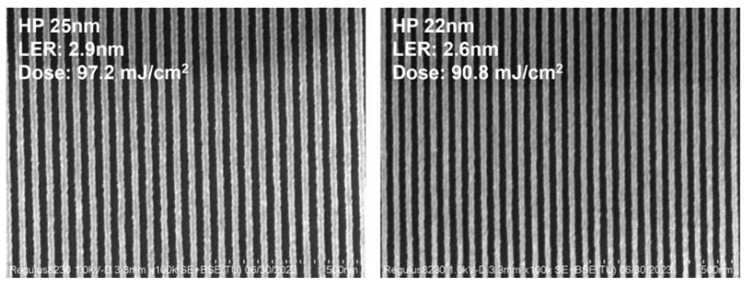
SEM images of 25 and 22 nm HP patterns by EUV lithography on n-CAR resist based on biphenyl iodonium perfluoro-1-butanesulfonate-modified polystyrene with a naphthalimide scaffold. Copyright 2024 Royal Society of Chemistry. Reproduced with permission from [55].

As previously noted, HfO_2_ has superior chemical and thermal stability and is resistant to oxidation, in contrast to organic polymers. Furthermore, due to their diminutive size (diameter under 1 nm), they demonstrate little light scattering and are applicable in both light-based lithography processes, namely DUV and EUV [54]. Figure 3 [55] shows the SEM images of 25 and 22 nm half-pitch patterns obtained by EUV lithography on a novel non-chemically amplified resist (nCAR) based on biphenyl iodonium perfluoro-1-butanesulfonate-modified polystyrene with a naphthalimide scaffold.

Enhancement of sensitivity by the incorporation of a metal sensitizer was noted with both EB and EUV methods. The enhancement in sensitivity arises from increased acid yield and electron efficiency, rather than elevated EUV photon absorption. Metal sensitizer salts significantly influence acid yield and dissolution properties. By carefully selecting a suitable sensitizer and optimizing its concentration, resist performance can be effectively enhanced. The incorporation of the sensitizer resulted in a 43% enhancement in sensitivity alongside a decrease in roughness. The enhancement in sensitivity is mostly attributable to the increased acid yield and electron efficiency [56].

A study investigating the stability of these clusters using spectroscopic techniques found that Zn-based oxoclusters, such as Zn(MA)(TFA), which are characterized by methacrylate and trifluoroacetate ligands, have labile ligands that undergo structural changes over a two-month period when kept as crystalline powder [57]. In contrast, when deposited as a thin film, these clusters undergo polymerization and/or hydrolysis within a few hours under ambient conditions. Nonetheless, the thin films exhibit stability in both air and vacuum for a sufficient duration for intact lithography applications and processing. The results elucidate the stability of resist systems, a crucial factor in developing new hybrid photoresists, and underscore that inorganic resists are prone to structural alterations that must be regulated to achieve reproducibility in lithographic performance [58].

### 2.3. Advantages and Limitations

To prolong Moore’s Law while managing stochastic effects to acceptable levels, three critical parameters are aerial picture contrast, photoresist, and dosage. The innovative EUV system incorporates 0.55 NA projection optics, as opposed to the 0.33 NA of the last generation. One primary effect of the elevated numerical aperture (NA) is that the aerial image contrast on the wafer is enhanced when fabricating identical structures. The 0.55 high-NA system enhances contrast, and with modifications to the mask absorber, the contrast can be preserved while reducing resolutions. The extensive transmission and accelerated phases guarantee that the productivity of the high-NA scanner is sustained even at elevated dosages. The primary advantage of the increased numerical aperture is the resultant enhanced contrast [59].

Two critical lithography challenges, line width roughness (LWR) and pattern collapse, have been recognized as necessitating enhancement as extreme ultraviolet (EUV) technology advances toward high-volume production. Pattern collapse constitutes a critical challenge in EUV lithography, constraining the process window and resolution. Reducing the resist thickness and aspect ratio of the resultant images might alleviate collapse and enhance resolution; nevertheless, it produces images that frequently lack sufficient durability for contemporary etch and integration processes [60].

The efficacy of EUV must be enhanced at the wafer level to achieve cost-effectiveness in technology. The efficiency of the current EUV source power throughput is inadequate. To incorporate working integrated circuits, the defect density on reflecting masks must be reduced. The utilization of non-pellicle masks presents significant challenges, leading to their replacement with movable pellicle and thermophoretic protection [52].

## 3. Electron Beam Lithography

The enhancement of resolution is recognized as the primary challenge in the advancement of optical lithography. Specific issues, such as resist outgassing and line edge roughness (LER), are encountered by Extreme Ultraviolet Lithography (EUVL), limiting its effectiveness. The absorption of extreme ultraviolet light by resist materials is also seen as a constraint on the application of EUV lithography.

In contrast, electron beam lithography (EBL) is employed as a sophisticated maskless technique for creating intricate patterns at the nanoscale, which is difficult to achieve with traditional methods [61]. Since its introduction in the 1970s, significant advancements have been made in EBL. Direct patterning is enabled by this technique without the need for a mask, unlike conventional mask writing procedures [62].

The highest practical resolution in EBL is achieved due to the short wavelengths associated with electrons, which can be less than 0.1 nm for energies between 10 and 50 keV. The resolution in EBL is primarily influenced by the quality of the resist used, requiring that the electron beam’s diameter be smaller than the dimensions of the final structure to attain enhanced resolution [63].

A concentrated electron beam is employed by EBL to delineate patterns on a substrate. The short wavelength of the electron beam enables a resolution of approximately 10 nm, free from diffraction limitations. A film or resist that is sensitive to electrons is patterned by applying it to the sample using a spin coating process, typically at speeds ranging from 1000 to 6000 rpm, to achieve a uniform layer [64]. When exposed to the electron beam, the resist’s solubility is modified, enabling it to become selectively more soluble in either the exposed or unexposed areas, depending on the type of resist utilized. The removal of the resist is accomplished by immersing it in a solution known as a developer [65].

An electron gun is a device designed to generate, accelerate, focus, and direct a beam of electrons toward a substrate. The process begins with the emission of electrons from cathodes or electron sources. These emitted electrons are subsequently accelerated by electrostatic fields, which enhance their kinetic energy and form them into a high-energy beam. Beam optics, comprising electric and magnetic focusing lenses along with a deflection system, are crucial for directing the beam toward a specific target on the substrate [66]. Traditionally, the beam is regarded as possessing a Gaussian profile, with the beam size defined as the full-width half maximum (FWHM) of the Gaussian distribution. The beam form is elliptical rather than circular; hence, it is more precisely characterized by the FWHM of a Gaussian distribution along the X and Y axes. In typical practice, the beam size and shape are improved by either focusing to create a sharp image of a resolution target or by performing a focusing process [67,68]. The effective generation and transmission of an electron beam to the substrate necessitate a high-vacuum environment.

**Figure 4:**
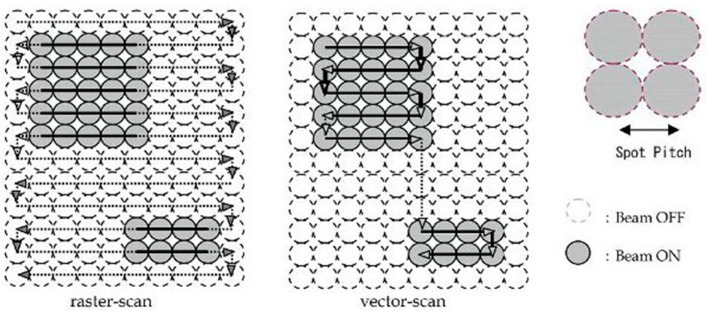
Comparison between raster and vector scanning methods in EBL. Copyright 2010, Cen Shawn Wu, Yoshiyuki Makiuchi and ChiiDong Chen. Originally published in [70] under https://creativecommons.org/licenses/by-nc-sa/3.0/ license (accessed on 20 January 2025). Available from DOI: 10.5772/8179.

Two primary techniques are employed for pattern generation in EBL: raster scanning and vector scanning. In a raster-scanning system, the exposing beam is directed in one direction at a consistent rate while the substrate is moved beneath it by a controlled stage. To form a patterned design, the electron beam is turned on and off millions of times during each scan, similar to the raster scanning method used in televisions [69]. Throughput is enhanced by the vector-scanning technique, which directs the exposure beam exclusively to the areas of the substrate that need exposure. A comparison between raster and vector scanning methods is shown in Figure 4 [70].

The interaction between electrons and solid materials, such as electron beam resist, involves various scattering events. During this interaction, two types of scattering occur: small angle scattering, also known as forward-scattering, which causes an increase in the diameter of the initial beam, and large angle scattering, or back-scattering, which results in the proximity effect. This proximity effect arises when the dose received by a specific pattern feature is affected by electrons that scatter from nearby features [71]. Backscattering occurs as electrons penetrate through the resist and into the substrate. During these interactions, primary electrons lose energy, leading to the generation of secondary electrons with energies ranging from 2 to 50 eV [72].

The exposure process of the resist is primarily attributed to the interaction of electrons with the material. Since the range of these interactions in the resist is limited to a few nanometers, their contribution to the proximity effect is minimal. A shape is exposed by an electron beam writer through the discrete scanning of a focused electron beam across predefined primitives. Designers create CAD patterns that represent their devices.

A critical process in electron beam lithography (EBL) involves layout fracturing, which entails breaking down the layout pattern into multiple non-overlapping rectangles known as primitives. This decomposition is necessary before the pattern can be processed by an electron beam writer [73]. Understanding how a CAD pattern is converted into primitives allows for better control over the writing process. The exposure of a shape is achieved by scanning the focused electron beam across the pattern, resulting in a shot pattern that fills the shape. Typically, the spacing between each shot is kept smaller than the beam size, ensuring solid exposure of the shape. Each shape is exposed individually until the entire pattern is completed [74].

### 3.1. Electron Beam Lithography Resolution

The ultimate resolution of electron beam lithography (EBL) is influenced not by the capabilities of electron optical systems, which can achieve resolutions down to 0.1 nm, but rather by the characteristics of the resist and the subsequent fabrication process. It was previously thought that this limit was dictated by electron scattering within the resist and possibly by the properties of the resist itself. There are two main types of scattering that can be analyzed separately and represented using Gaussian distributions. The first phenomenon, known as forward-scattering, involves minor angle inelastic scattering of electrons as they penetrate the resist, characterized by a narrower distribution. For thin resists measuring less than 0.1 µm and under high accelerating voltages exceeding 100 kV, this width becomes negligible, around 1 nm.

**Figure 5:**
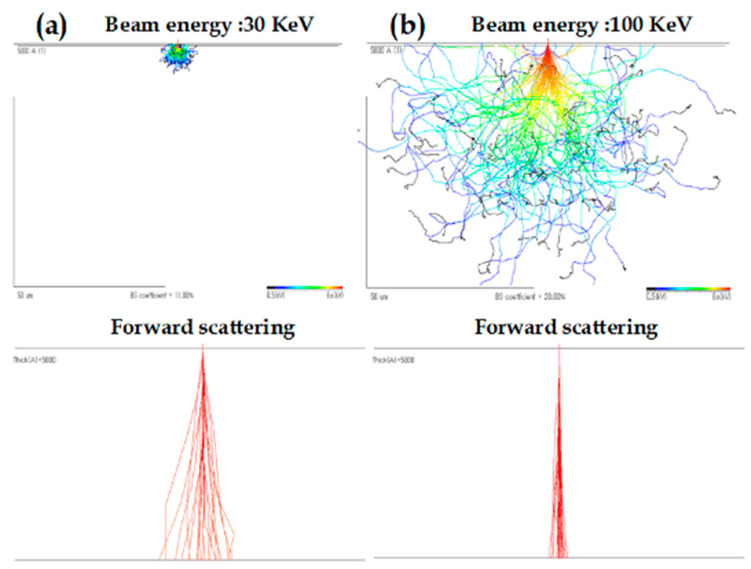
Monte Carlo simulation of electron forward-scattering for (**a**) 30 keV and (**b**) 100 keV beam energies. Forward scattering is higher at beam energy of 30 keV than 100 keV. Copyright 2010, Cen Shawn Wu, Yoshiyuki Makiuchi and ChiiDong Chen. Originally published in [70] under https://creativecommons.org/licenses/by-nc-sa/3.0/ license (accessed on 20 January 2025). Available from DOI: 10.5772/8179.

The second phenomenon, referred to as back-scattering, involves significant angle elastic scattering primarily occurring in the substrate beneath the resist. In this case, electrons are reflected from the substrate back into the resist layer. The total exposure contributed by back-scattered electrons is roughly equivalent to that from incoming electrons but is spread over a diameter that corresponds to the electron range within the substrate [75]. Figure 5 [70] shows the Monte Carlo simulation of electron forward-scattering for different beam energies of 30 keV and 100 keV. It can be seen that forward-scattering is much higher at 30 keV than 100 keV. The resist sensitivity is observed to decrease with higher accelerating voltages, while lower accelerating voltages are advantageous from a throughput perspective. However, this comes at the cost of reduced resolution. An effective strategy to mitigate this limitation is the use of a thin imaging layer [76].

Using electron beam lithography (EBL), high-performance CMOS devices with a gate length of 36 nm have been successfully fabricated. EBL has demonstrated effectiveness in fabricating fully scaled 0.5 µm CMOS devices, as well as 0.25 µm NMOS and PMOS devices. Additionally, it has been utilized for the production of 100 nm T-gate GaAs PHEMTs and single electron tunneling (SET) devices, all known for their capabilities in high-density, low-power memory applications [77].

### 3.2. Electron Beam Lithography Resists

PMMA (polymethyl methacrylate) is the primary resist employed in electron beam lithography due to its excellent contrast and resolution. It can function as both a positive and negative resist throughout the fabrication process. When exposed to an electron beam, PMMA undergoes chain scission, leading to the formation of smaller fragments that are subsequently removed by developers such as diluted methyl isobutyl ketone (MIBK) and a mixture of isopropyl alcohol (IPA) and deionized water, thus acting as a positive tone resist.

If PMMA is subjected to high doses exceeding 10 mC/cm^2^ at 30 keV, crosslinking occurs, resulting in a dense molecular network that exhibits resistance to developers and functions as a negative tone resist. Despite its advantages, PMMA has limited dry etch resistance, which can impede high-throughput applications. This resist has been extensively utilized in various applications, including electronic devices, nanoimprinting, photon sieves, masks, metal nanowires, and quantum wire production. Studies indicate that while PMMA remains the preferred choice for electron beam lithography due to its favorable properties, ZEP has emerged as a strong alternative [78].

**Figure 6:**
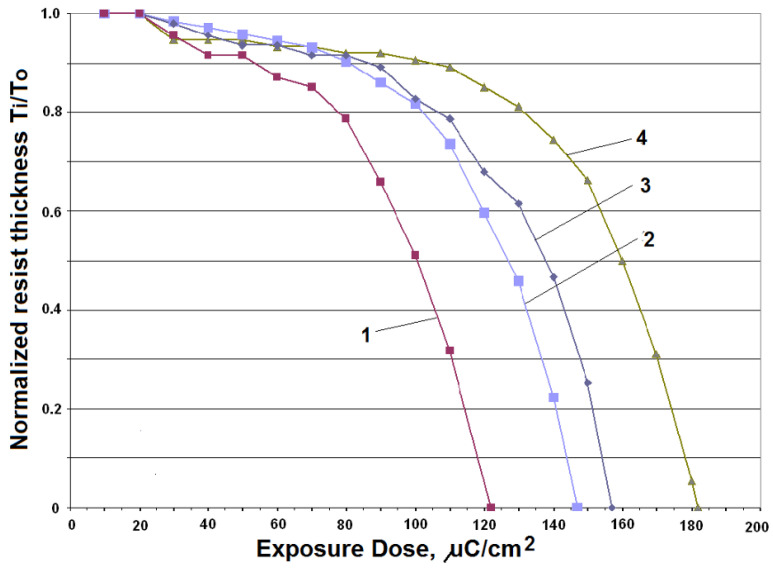
Different contrast curves for PMMA on silicon using EBL. Copyright 2018 IOP Publishing. Reproduced with permission from [79]. All rights reserved.

**Figure 7:**
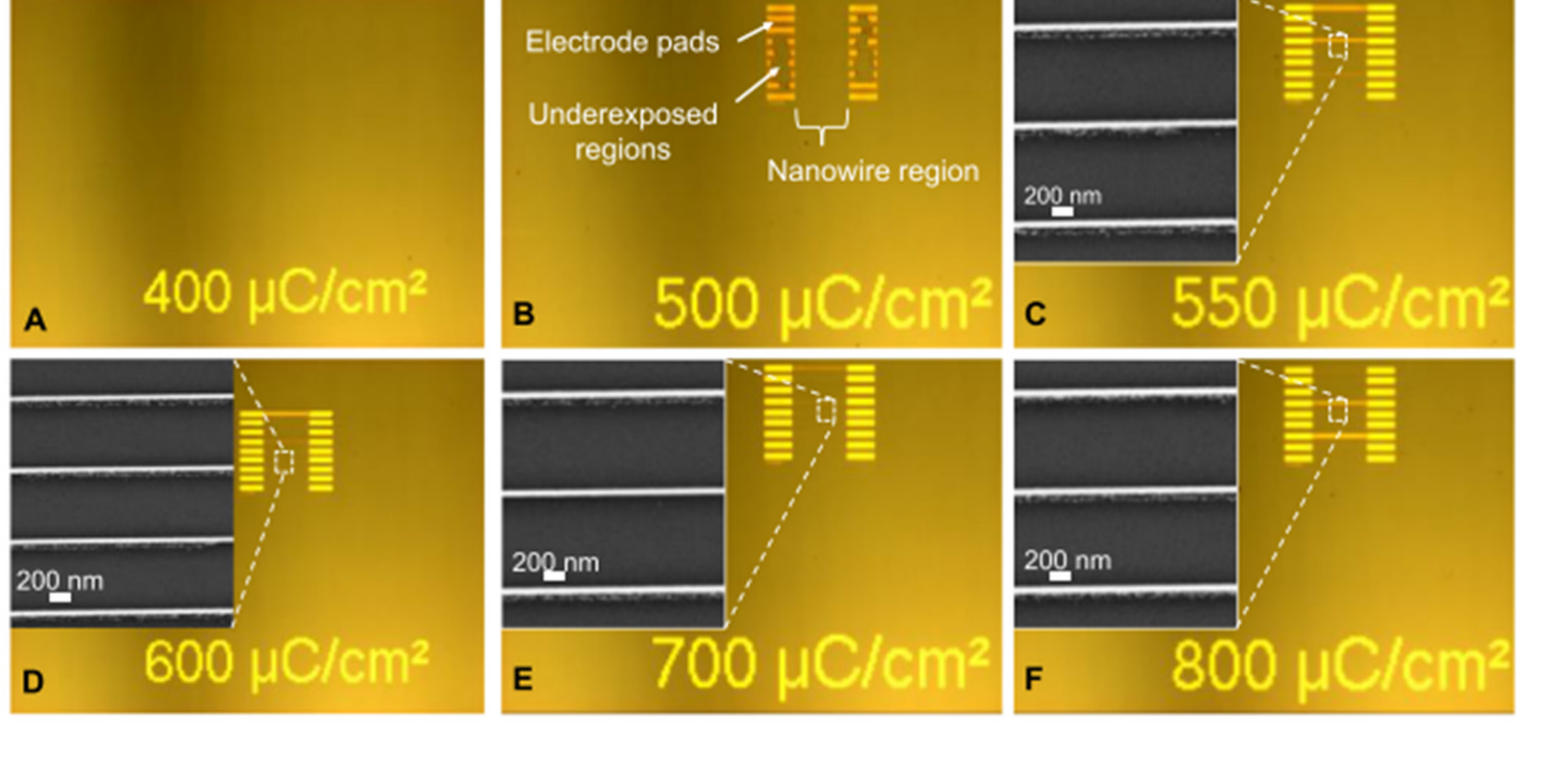
Optical microscopy and SEM images of metal nanopatterns fabricated with EBL with a dose range varying from (**A**) 400 μC/cm^2^, (**B**) 500 μC/cm^2^, (**C**) 550 μC/cm^2^, (**D**) 600 μC/cm^2^, (**E**) 700 μC/cm^2^, (**F**) 800 μC/cm^2^. Copyright 2024 IOP Publishing. Reproduced with permission from [80]. All rights reserved.

Figure 6 [79] shows the contrast curves for PMMA for different resist thickness, different developer solutions, and different doses to clear the resist. Curve 1 shows an initial PMMA thickness of 300 nm, with a developer of H_2_O:IPA 3:7 solution, with a dose to clear = 122 μC/cm^2^; Curve 2 shows an initial PMMA thickness of 1134 nm, with a developer of H_2_O:IPA 3:7 solution, with a dose to clear = 147 μC/cm^2^; Curve 3 shows an initial PMMA thickness of 300 nm, with a developer of MIBK:IPA 1:3 solution, with a dose to clear = 157 μC/cm^2^; Curve 4 shows an initial PMMA thickness of 1165 nm, with a developer of MIBK:IPA 1:3 solution, with a dose to clear = 182 μC/cm^2^. Varius optical microscopy and SEM images of metal nanopatterns fabricated using EBL with different dose ranges are shown in Figure 7 [80].

Chemically amplified resists (CARs) have become prominent as effective materials for lithography due to their high sensitivity and resolution. Typically, CARs are composed of an acid-reactive polymer, often an epoxy-based polymer, in combination with a photoacid generator (PAG). A newly developed PAG-bound polymer that incorporates glycidyl methacrylate (GMA), methyl methacrylate (MMA), and triphenyl sulfonium salt methacrylate (TPSMA) has been utilized as a negative tone resist in electron beam lithography (EBL) [81]. However, CARs encounter challenges related to the release of photoacid when exposed to an electron beam, which serves as a catalyst and influences sensitivity.

To overcome this limitation, a novel MAPDST–MMA copolymer that includes the sulfonium group has been developed as a non-chemically amplified negative resist (n-CAR) for advanced lithography applications. This innovative material exhibits exceptional sensitivity to electron beam radiation, achieving high-resolution features down to 20 nm. The MAPDST–MMA copolymer demonstrates impressive performance, achieving a resolution of 20 nm for a 1:2 line/space ratio and maintaining low line edge roughness (LER) between 1.8 and 2.3 nm. Furthermore, this resist material shows strong etch resistance against plasma etch chemistry [82].

Several new resist materials have been developed using radiation-sensitive sulfonium groups, with MAPDST serving as the primary ingredient. These materials were polymerized with methyl methacrylate (MMA), 4-carboxy styrene (STYCOOH), or N-vinylcarbazole (NVK) as co-units through a free-radical polymerization process [83]. Sodium PSS, or water-soluble poly (sodium 4-styrenesulfonate), can function as a negative electron beam blocker and is produced using water. Due to the presence of metal sodium, PSS exhibits significantly greater resistance to dry etching compared to PMMA. However, it is important to note that sodium PSS lacks the sensitivity and contrast of organic resists like PMMA, limiting its use in patterning thick or high-resolution structures. Despite these limitations, feature sizes for sparse patterns down to 40 nm have been achieved with this material [84].

Hydrogen silsesquioxane (HSQ) inorganic electron beam resists are recognized for their capability to achieve both high sensitivity and high resolution in lithographic applications. They demonstrate sensitivity levels comparable to chemically amplified resists (CARs) while providing excellent contrast. Features as small as 15 nm have been successfully written with a line width roughness (LWR) of 1.9 nm. Additionally, HSQ has shown to etch slowly compared to thermally grown SiO_2_ during reactive ion etching [85]. When utilized in electron beam lithography (EBL), HSQ can reach resolutions of approximately 5 nm. Despite its effectiveness, the low sensitivity of HSQ often limits its application in various scenarios. While HSQ is considered one of the best EBL resists due to its performance, it is not as sensitive as some organic resists like PMMA.

Another category of resist includes water-based conducting polyanilines, which also fit within this group. Furthermore, eco-friendly options such as biomaterials derived from silk fibers have been explored as potential EBL resists [86]. Using 3D EBL techniques, functional and arbitrary 3D nanostructures have been fabricated with precision below 15 nm using only water as a medium. The innovative approach of genetically engineering recombinant spider silk proteins as a resist allows for the creation of any 3D object at the nanoscale with high resolution and strength. This method enables the precise shaping of polymorphic spider silk proteins at nearly the molecular level by quantitatively describing structural transitions induced by energetic electrons at varying depths within the 3D protein matrix [87]. Table 1 shows advanced in resist materials and improvement in resolution with EBL.

### 3.3. Advantages and Limitations

A focused electron beam serves as the most precise writing tool available, capable of creating pattern features as small as a few nanometers. Unlike traditional photolithography, electron beam lithography (EBL) does not require a pre-existing pattern mask; it can directly write patterns based on stored data. EBL remains the preferred method for producing nanometer-scale objects in small quantities due to its high resolution and flexibility in pattern modification [90]. This technique surpasses traditional photolithography, which is limited by diffraction effects that restrict spatial precision to a few hundred nanometers. Although EBL does have a diffraction limit, the high-energy electrons utilized in various studies enable a diffraction limit in the nanometer or even picometer range. Research has demonstrated that EBL can achieve isolated feature sizes down to 2 nm and half-pitch dimensions of 5 nm using advanced materials like hydrogen silsesquioxane (HSQ) resist. The resolution capabilities of EBL are primarily constrained by factors such as electron scattering within the resist and imperfections in the electron optics, rather than by the size of the electron beam itself [91].

The biggest task for electron beam lithography is to increase throughput. A single fine beam has struggled to achieve both higher sharpness and higher throughput simultaneously. While optical lithography can design a wafer in minutes, several tens of hours are required for EBL to accomplish the same task due to its serial process. To address this issue, variable shaped beam systems and character/cell projection methods have been developed. However, the throughput of the most advanced systems has not yet reached the necessary levels [76]. As discussed in earlier sections, electron scattering (both forward- and back-scattering) limits the resolution of EBL. Primary electrons are more likely to move forward when the acceleration voltage of the electron beam is low, as lower voltages enhance forward-scattering. The proximity effect, caused by back-scattered electrons that lead to increased exposure, further deteriorates clarity. Additionally, secondary electrons with energies ranging from 2 to 50 eV contribute to reduced resolution. These challenges make EBL less practical compared to other methods. To circumvent these issues, new next-generation lithographic techniques such as X-rays and ion-beam lithography are being developed.

## 4. X-Ray Lithography

X-ray lithography (XRL) was developed in the early 1970s to enhance the resolution of existing lithography techniques used in the semiconductor industry. At that time, UV projection lithography was the leading technology, capable of resolving features down to one micron. However, as the microelectronics industry aimed for smaller sizes, specifically around 250 nm, UV lithography was perceived as having limited future prospects due to its inability to overcome the physical limits imposed by diffraction. To align with Moore’s Law and the International Technology Roadmap for Semiconductors (ITRS), there was a clear need for radiation with shorter wavelengths to facilitate further miniaturization of devices [92].

H.I. Smith and Spears from MIT Lincoln Labs were among the first to propose X-ray lithography (XRL) in the 1970s [93]. Following this, Bell Laboratories and various U.S. industrial organizations developed X-ray systems utilizing palladium (Pd) targets [94]. The wavelengths of radiation employed in XRL range from 0.4 nm to 5 nm. XRL is also the initial step in the LIGA process, which involves placing metal electrodes on the created resist structure to form a mold or electrode for subsequent replication processes, including electro discharge machining or molding.

**Figure 8:**
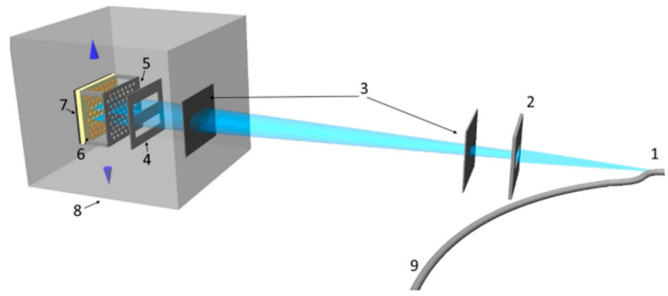
Schematic diagram of a LIGA X-ray beamline. 1: radiation area; 2: front-end absorber. 3: beryllium windows; 4: beam stop and spectral filter; 5: X-ray mask; 6: substrate; 7: motorized translation stage; 8: exposure chamber; 9: electron beam orbit. Copyright 2021 IOP Publishing. Reproduced with permission from [96]. All rights reserved.

There are two categories of X-rays: soft and hard. Soft X-rays have energy levels between 150 eV and approximately 2 keV, while hard X-rays possess energies exceeding 5 keV. Soft X-rays are effective for high-resolution designs requiring less resist thickness. Deep X-ray lithography (DXRL) is frequently utilized in the LIGA process and for heating thick resists [95]. A schematic diagram of a LIGA X-ray beamline is presented in Figure 8 [96].

Various applications of X-ray lithography (XRL) include IBM’s fabrication of 64 MB DRAMs utilizing XRL for the gate level, Mitsubishi’s development of a 1 GB DRAM test site featuring 0.14 µm gates and a stacked capacitor with a capacitance exceeding 25 fF/cell, and a collaboration between Toshiba and NTT to create a 4 GB DRAM test site with functional cells at a pitch of 0.24 µm. Additionally, IBM has produced CMOS logic devices, including a fully functional 64 KB SRAM with 0.2 µm features, while NTT has fabricated CMOS logic devices featuring a 12 KB SRAM and a 48 × 48 bit multiplier, also with 0.2 µm features. Motorola has developed CMOS logic devices, including a fully functional 1 MB SRAM, using three levels of XRL with feature sizes of 0.375 µm. Furthermore, IBM has fabricated CMOS test circuits, including ring oscillators with feature sizes of 0.1 µm [97]. Silverman et al. [97] successfully created a polysilicon gate measuring 50 nm by etching a resist line that was 70 nm wide.

X-ray lithography (XRL) involves transferring patterns to a wafer substrate using a mask and a highly collimated X-ray beam. An X-ray radiation system comprises several components: a high-power X-ray source, a beamline, a mask, a substrate coated with resist, and additional exposure elements. Key factors that contribute to the reliability of an XRL system include resolution, critical dimension (CD) control, and overlay accuracy. Historically, X-ray tubes served as sources of X-ray radiation; however, their large spot size and lower beam intensity rendered them inadequate for advanced technological requirements. To address these limitations, plasma sources were introduced, offering higher radiation intensity with smaller spot sizes and beam diameters [98,99]. In the late 1970s, the Naval Research Laboratory conducted experiments demonstrating the effectiveness of plasma-generated X-ray sources for lithographic applications [100].

There are two primary types of plasma sources used in this field: laser-induced plasma sources and discharge plasma sources [101,102]. While discharge plasma sources provide significantly higher radiation intensity, laser plasma sources have lower intensity levels. At that time, the absence of affordable, high-power lasers led to the widespread use of discharge plasma sources. However, the adoption of plasma sources for X-ray radiation has diminished due to challenges such as electrode erosion and damage to masks and wafers caused by these sources [52].

Recent advancements in X-ray sources have led to the development of synchrotron radiation sources, which generate high-intensity X-rays using magnetic storage rings. In these rings, electrons emit a highly collimated beam of synchrotron radiation, achieving a maximum depth of focus of approximately 0.3 mm [103]. The initial lithography experiments utilizing synchrotron radiation were conducted in 1974 by Spiller and Feder [104]. By 1980, the X-ray resonator at IBM Yorktown was exploring the potential of using storage rings as X-ray sources.

Over several years, IBM established an X-ray lithography (XRL)-dedicated beamline at Brookhaven National Laboratory. In 1991, IBM constructed an Advanced Lithography Facility in East Fishkill, New York, to accommodate the HELIOS superconducting storage ring, which was provided by Oxford Instruments. The ring operated with a small injector at approximately 200 MeV and reached 686 MeV at 165 mA. With standard resist materials, the X-ray flux from HELIOS, which had an effective wavelength of around 0.8 nm, could undergo a second exposure. Throughout the 1990s, IBM expanded its X-ray initiatives, including the production of high-resolution DRAM chips that showcased the capabilities of XRL [105].

The radiation produced by a synchrotron radiation source travels through an ultrahigh vacuum beamline [106]. Given that the length of a typical beamline can extend to several tens of meters, electromagnetic waves can be approximated as planar at the surface of the X-ray mask membrane. Throughout the beamline, the radiation interacts with various optical elements arranged in distinct configurations. The beam passes through a series of foils made from different materials and strikes one or more mirrors, which are crucial for selecting the appropriate wavelength spectrum before reaching the mask [107]. This process occurs while accounting for the unavoidable scattering of X-rays caused by residual gas molecules present in the beamline [92]. To enhance X-ray diffraction, a stepped attenuator is employed, which increases in thickness as the distance from the optical axis of the X-ray lens grows. This attenuator, made from a 100 μm thick polyimide sheet, is placed in front of the X-ray mask. By varying the dose through attenuators of different thicknesses and splitting the X-ray beam used for structuring, new properties can be imparted to the resulting microstructures [108].

### 4.1. X-Ray Lithography Resolution

The creation of a photoelectron, which possesses energy equal to the X-ray energy minus the binding energy of the electron to its atom, represents the fundamental interaction that occurs when a low-energy X-ray is absorbed by a solid. During the resist process, over ninety percent of the emitted photoelectrons originate from an inner shell, with their binding energy typically in the range of several hundred volts. After the photoelectron is ejected, the atom remains in an excited state. This excited state can decay either by emitting another photon, known as fluorescence, or by releasing an Auger electron [109]. Historically, it was believed that the resolution of X-ray lithography was constrained by the maximum range of the photoelectrons and Auger electrons released upon X-ray absorption [110].

**Figure 9:**
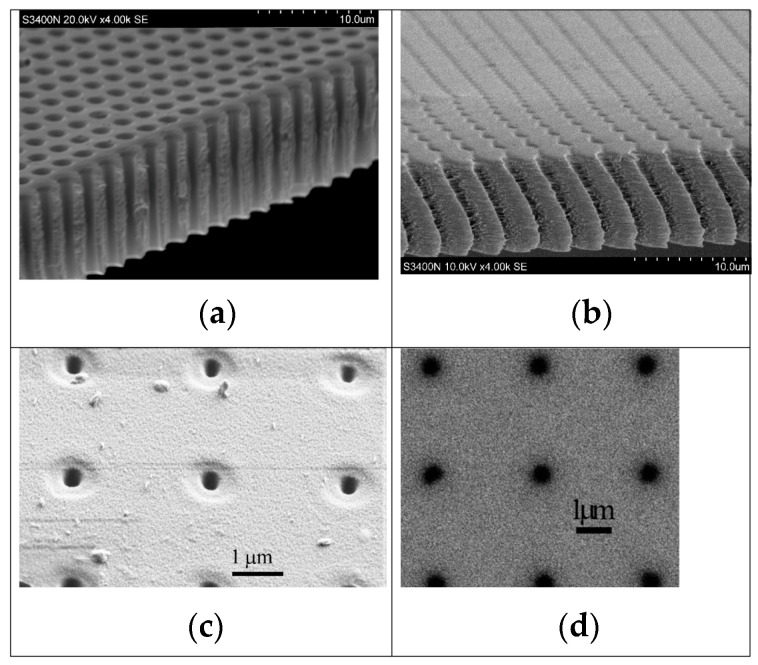
(**a**–**c**) SEM images of channels fabricated in PET film using X-ray lithography and (**d**) X-ray mask. Copyright 2021 IOP Publishing. Reproduced with permission from [96]. All rights reserved.

When employing X-ray lithography (XRL), the typical resolution achieved is around 15 nm, with minimal false scattering occurring in the process. Figure 9 [96] illustrates SEM images of channels fabricated using XRL in polyethylene terephthalate (PET), a material with low sensitivity. In the images, Figure 9a,b exhibit aspect ratios of approximately 7 and 20, respectively. In lithography, particles such as ultraviolet photons and electrons often experience significant spurious scattering, resulting in unintended exposure in areas that should remain unaffected [111]. Soft X-rays, particularly those in the energy range of 140 to 500 eV, are favored because its mask is easier to fabricate compared to hard X-rays. Hard X-rays need thick metal to block it, and it is difficult to make such a mask with very narrow line width through a very thick metal film. The application of soft XRL is suitable for achieving high-resolution structures. Soft X-rays, particularly those in the energy range of 140 to 500 eV, are favored as they provide a satisfactory balance between diffraction-limited resolution and resolution limited by photoelectron blur and penumbral effects [95].

### 4.2. X-Ray Lithography Resists

To successfully transfer patterns using X-ray lithography, a radiation-sensitive organic substance known as resist is required. To achieve optimal performance in the X-ray lithography (XRL) process, the resist material must possess several key characteristics, including submicron resolution of less than 0.1 µm, a sufficient aspect ratio, excellent thickness uniformity, thermal stability, minimal defects, strong contrast, and tolerance to dosage variations. The sensitivity of positive tone/chain scission-type resists to X-rays is relatively low, typically falling between 100 and 2000 mJ/cm^2^ at a wavelength of 8 Å [112]. In contrast, negative resists have been developed that exhibit sensitivities around 10 mJ/cm^2^ at a wavelength of 4.37 Å [112,113]. A comparatively thin layer of resist is required to obtain a consistent X-ray exposure through the resist thickness. This is because the X-ray absorption is essentially linear with thickness when the resist layer is relatively thin. Certain operations necessitate the presence of resist layers that are as thick as 3 μm. The need for uniform absorption in this scenario is a relatively short wavelength, which is less than 10 Å. To enable operation at longer wavelengths, it is essential to utilize a thin resist layer along with one or more layers of etch-resistant thin films [114]. Resist sensitivity is proportional to absorption coefficient α, higher absorption results in higher sensitivity, and absorption coefficient α depends greatly on wavelength.

X-ray lithography and electron beam lithography are two methods that are extremely similar in terms of how they expose polymer films. In both instances, the energy of the particles when they collide with one another is significantly greater than the energy that is necessary to either make or break a chemical bond. The interaction between X-rays and the resist is significantly less powerful than that of electrons. Following the absorption of an X-ray photon, a shower of secondary electrons is produced. These electrons carry the majority of the energy from the Incident photon, leading to significantly stronger interactions with the resist material. It can be inferred that secondary electrons are primarily responsible for the chemical transformations occurring within the resist. Essentially, an X-ray exposure of a resist can be viewed as an electron exposure. The key distinction between an X-ray exposure system and an electron beam exposure system lies in the energy levels: incident electrons in an electron beam system typically range from 10 to 50 keV, while secondary electrons generated by soft X-ray exposure possess much lower energies. Consequently, any electron beam resist can also function as an X-ray resist [115].

**Figure 10:**
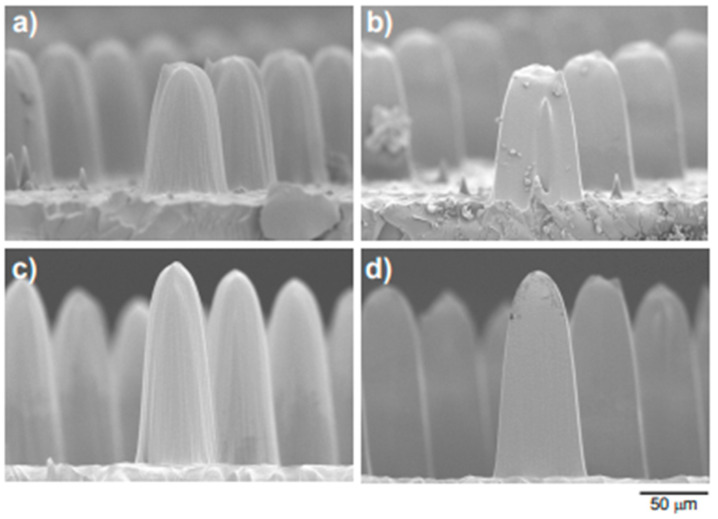
Cross-sectional SEM images of cone (**a**,**c**)- and pyramid (**b**,**d**)-shaped Si absorbers transferred to a PMMA structure using XRL. Copyright 2015 MDPI. Reproduced with permission from [116].

Polymethyl methacrylate (PMMA) is widely recognized as one of the most effective resists for X-ray lithography due to its ability to achieve the highest resolution among all resists, reaching down to 100 Å. However, PMMA’s sensitivity is relatively low, around 1000 mJ/cm^2^, and it exhibits poor etch stability. Figure 10 illustrates cross-sectional SEM images of cone- and pyramid-shaped silicon absorbers that were transferred to a PMMA structure using X-ray lithography [116]. The IBM Research XRL program successfully utilized novolak positive resist, achieving linewidth control of 7 nm on device wafers at a dosage of 700 mJ/cm^2^. Most device processing has been confirmed to be compatible with this resist material [117].

It is highly advantageous for future lithography technologies to utilize standard novolak-based resists (dissolution inhibitor/matrix resin) due to their demonstrated stability across various applications. Unfortunately, when synchrotron radiation sources are processed similarly to optical lithography, their sensitivity to X-rays falls short by at least a factor of ten. However, if the processing of the resist, especially during the development phase, is optimized for X-ray lithography, certain optical novolak/diazotype resists can yield favorable results regarding resolution and stability, even with increased sensitivity. The development step plays a crucial role in this optimization. It was determined that HPR 204 and HUNT WX 214 produced the most favorable outcomes in terms of resolution and stability. Additional chemical amplification was implemented later in order to further enhance the sensitivity of novolak resists [118].

The formulation of a dual tone resist that was devised based on acid-catalyzed deprotection was accomplished by combining poly (t-butoxycarbonyloxystyrene) (PBOCST) with triphenylsulfonium hexafiuoroantimonate (15 weight percent of the total solid) in Arcosolv PM Acetate. Irradiation of the onium salt results in the production of strong Bronsted acids, which are responsible for catalyzing the transformation of the lipophilic polyhydroxystyrene (PBOCST) into the hydrophilic (even acidic) polyhydroxystyrene (PHOST).

At the lithography beamline of the Stanford Synchrotron Radiation Laboratory (SSRL), where the incident dosage necessary for achieving a final thickness of one hundred percent was thirteen millijoules per square centimeter, the sensitivity characterization was carried out. In the range of 15 to 20 percent, the resist contrast that was obtained was extremely high [119]. Further down the line, excellent results were obtained by various research groups in a number of commercially available resists, such as Apex-E, UVII-HS, UV-4, TDUR-N908, and SAL601 [97]. Different types of X-ray lithography resists and their resolution are shown in Table 2.

### 4.3. Advantages and Limitations

When applied to thick, single-layer resists with a high aspect ratio and depth of focus, X-ray lithography (XRL) can pattern features with resolutions of 15 nm or less while maintaining a high level of fidelity. The wavelength of X-rays is under 10 nm, resulting in a relatively modest diffraction limit. This characteristic allows XRL to circumvent the resolution limitations posed by diffraction that are encountered in EUV lithography. Additionally, XRL benefits from a substantial number of experienced vendors who have developed XRL tools and possess a deep understanding of the technology, its challenges, and the necessary tooling requirements, along with a considerable base of knowledgeable end users [122].

However, mask stability presents a significant challenge for XRL systems, particularly concerning radiation damage that accumulates over the mask’s operational life.

Recent studies indicate that under extreme conditions, distortions of around 20 nm can be observed. The masks used in XRL are particularly vulnerable to damage or bending due to their thinness [123]. Concerns regarding device radiation damage during X-ray lithography have also been raised by researchers in this area. While X-ray synchrotron sources can generate high-power X-rays, they come with substantial maintenance costs and physical space requirements [124]. Consequently, other advanced lithographic techniques are being explored.

## 5. Ion Beam Lithography

Ion beam lithography (IBL) is an advanced development in the field of nanofabrication due to its potential to achieve high-resolution patterning beyond the capabilities of traditional optical lithography. One of the key advantages of IBL is its ability to bypass the diffraction limit, which has constrained optical methods. The ability to produce patterns smaller than 100 nm marks a significant leap forward.

However, IBL faces challenges similar to those encountered in electron beam lithography, primarily the proximity effect but only for very light ions [125]. The lateral scattering of ions and the secondary electrons they generate can cause the beam to spread, reducing the precision of the patterning. This limits the ability to create very fine features with high resolution. To overcome these challenges, ongoing research focuses on improving ion beam control, resist materials, and techniques to minimize scattering and improve pattern fidelity. This in turn limits the spreading of exposure features in a resist to less than 10 nm [126].

**Figure 11:**
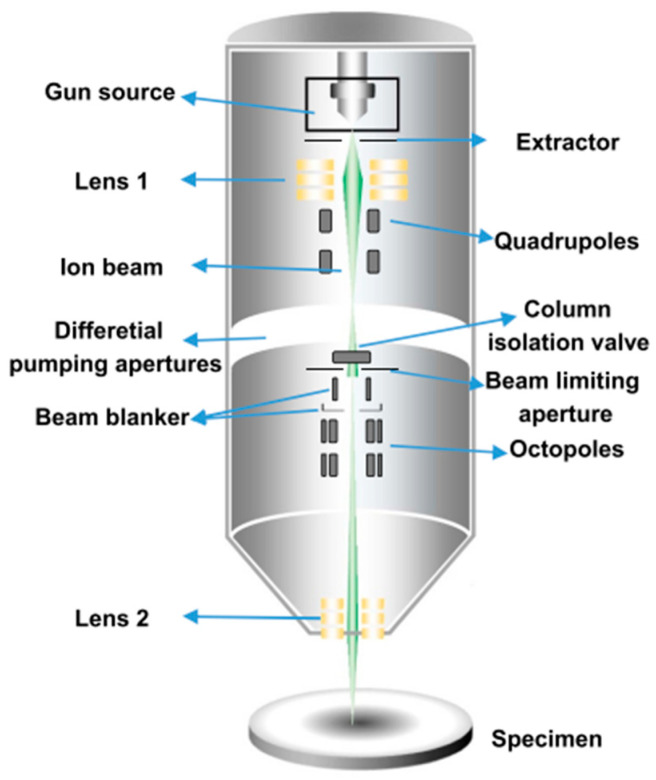
Schematic diagram of a helium ion beam system utilizing gas field ion source. Copyright 2021 IOP Publishing. Reproduced with permission from [127]. All rights reserved.

In most cases, the range of light ions in resist in the energy domain spanning from 50 to 150 keV is comparable to resist thickness. Ions are therefore effective at exposing resist, and the minimum dose required for exposure is between 10^12^ and 10^13^ ions per square centimeter. The range of light ions like H^+^ or He^+^ is typically very predictable and straight because of their relatively low mass and charge, and hence there is minimal lateral scattering. A schematic diagram of a helium in beam system utilizing a gas field ion source is shown in Figure 11 [127]. It is because of this that the dose at a particular location does not depend on the exposure of areas that are adjacent to it; in other words, there is not much of a proximity effect [128,129]. As a result of the fact that ion/electron collisions are the primary interaction for rapid light ions, it is necessary for an ion to undergo many collisions before it finally arrives to a state of permanent rest.

The major method of contact for ions is the transfer of momentum between the slow ions and the surface atoms. This results in the physical sputtering of the material, which causes the atoms to reorganize themselves and brings about structural changes. There are now three unique and independent ion beam techniques that can create structures. These methods are as follows: (1) Focused ion beam (FIB), which uses a finely focused ion beam (typically heavy ions like gallium at 30 keV) to locally sputter or deposit material on a surface and finds its use in microfabrication, milling, and modification of surfaces; (2) Proton beam writing (PBW), in which fast protons (generally MeV) are used to write precise, deep, three-dimensional patterns into a resist material; (3) Ion projection lithography (IPL), which involves projecting medium-energy ions (around 100 keV) through a patterned mask onto a surface, typically used for rapid production of microstructures [130].

### 5.1. Focused Ion Beam (FIB)

The introduction of focused ion beam (FIB) technology dates to the 1970s, when the source of ions was a standard implanter, and the beam diameter that was attained was in the range of 3 µm. In the time since then, it has become the most advanced and commonly utilized technique for ion beam lithography. There are three primary motivating factors that contribute to the effectiveness of FIB techniques in nanofabrication. The direct write approach, which combines milling, depositing, etching, and other processes into a single piece of equipment, is the first method that simplifies the sample preparation process and reduces total throughput. The ability to create 3D structures with a high aspect ratio is another significant benefit, especially in fields like microelectronics, materials science, and nanotechnology.

The high precision and control offered by FIB allow for detailed and intricate designs at the nanoscale, making it invaluable for things like device prototyping, failure analysis, and even sample preparation for tunneling electron microscopy (TEM). Finally, the nanoscale interaction volume is crucial in understanding how the ion beam interacts with the material. This volume typically ranges to few tens of nanometers for 5–50 keV gallium ions [131]. The ions induce various processes such as sputtering, deposition, and even changes in the material’s atomic structure within that interaction volume. This makes FIB not just a tool for material removal or structuring, but also one that can provide insight into the material properties at the microscopic and nanoscopic levels.

Patterns may be created in nearly any material using FIB, which is a unique feature in comparison to other lithography processes, which are restricted to patterning resist materials due to their limitations. On the other hand, the process is relatively sluggish, and a gallium ion source with a power of 30 keV can remove between one and ten atoms for every incident ion. Both the electrons and the atoms absorb energy from the incident ion, which causes the ion to lose energy. Due to Coulomb contact, the electrons in the material are excited to transition into bound states or continuum states when the ion passes through. On the other hand, the loss of atoms is partially attributable to a limited number of random collisions, which results in a large loss of energy during the event. Due to this, the ion is diverted away from its intended path, and the atom in the solid is moved away from its position in the lattice [132].

There has been a significant amount of progress made in the creation of ion sources for the operation of FIB. Earlier FIB instruments had a low current density and a low brightness of the ion sources, which resulted in poor efficiency and a limited application range. The liquid metal ion source has been the most popular option of FIB ion source ever since the introduction of the liquid gallium metal ion source. This source generates a brightness of around 10^6^ Acm^−2^Sr, making it the most preferred choice.

In addition, new ion sources are being developed, including gold, silicon, germanium, and other liquid ion sources. These new ion sources have the potential to improve the long-term stability of ion beams thanks to their characteristics. In addition, various ion sources, such as liquid metal alloy ion sources (LMAISs), have been utilized for specialized applications, such as high-resolution FIB implantation. These ion sources include AuSi, AuSiB, PdAs, PdAsB, NiB, and NiAs. Investigations are also being conducted on additional promising LMAIS candidates such as Ga_35_Bi_60_Li_5_ and Co_31_Nd_64_B_5_ [133].

The earlier FIBs that were integrated with an inductively coupled plasma (ICP) ion source could perform large-volume milling operations at a removal rate that was approximately one hundred times quicker than that of a Ga^+^ source. This was achieved by delivering up to two microamperes of Xe^+^ ions that were concentrated into a sub-five micrometer spot. Nevertheless, the bigger spot size that is produced by a plasma FIB source results in a reduction in the quality of the cross-sectional area, and the beam currents are not sufficiently high to satisfy the requirements of the advanced level [134].

Recent developments in the field of nanofabrication have been made possible by gaseous field ion sources (GFISs), which are capable of producing minuscule beam currents of less than 2 pA and producing a spot size of approximately 0.35 nm. For instance, with the advanced He^+^/Ne^+^ gas field ion source microscope resolutions can reach 0.25 nm, and feature sizes can be less than 5 nm, pushing the limits of nanofabrication. Although Ga^+^ ions are still used, their maximum penetration depth is limited to about 80 nm. Furthermore, their trajectory is no longer as directed downward, and they experience more back-scattering, which may affect the quality of the process.

When compared to gallium metal ions, the He^+^ beam has a significantly smaller spot diameter than a Ga^+^ beam, sometimes as small as 0.3 nm, which allows for higher-resolution imaging. This is important in nanoscale characterization because it enables finer details to be resolved. The larger penetration depth of He^+^ ions in PMMA (polymethyl methacrylate) can be advantageous for imaging or material modification, as it can interact deeper within the sample compared to Ga^+^ ions, which may have shallower penetration. Also, He^+^ ions do not contribute to any contamination [133].

**Figure 12:**
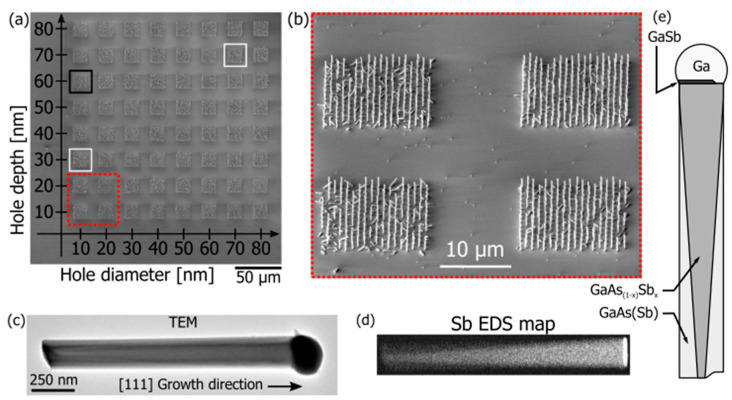
GaAsSb nanowires grown by varying different FIB conditions. (**a**) 8 × 8 hole depth -diameter matrix; (**b**) arrays of 18 × 15 nanowires; (**c**) TEM of broken defect-free nanowire with (**d**) corresponding Sb EDS map; (**e**) schematic of nanowire features including GaSb crystal in Ga droplet. Copyright 2023 IOP Publishing. Reproduced with permission from [138]. All rights reserved.

Recently, FIB was used for the bit milling of 2D and 3D nanostructures [135]. FIB-milled structures consisting of 40 nm thick segmented gold islands for surface plasmon polaritons and for resonant plasmons was demonstrated [136]. Through the use of FIB milling, regular and periodic lines measuring 200 and 300 nm in wavelength have been milled at 70 pA with a high degree of fidelity in PDMS structures. Another application of FIB was the fabrication of a field emitter tip in a diamond crystal, which had a radius of less than 100 nm [137]. Mosberg et al. [138] demonstrated the growth of self-catalyzed GaAsSb nanowires using different FIB patterning conditions as shown in Figure 12 [138]. In addition to ion implantation of doping species in semiconductors and targeted ion etching on a submicron scale, the other significant applications of FIB technology are scanning ion microscopy (SIM) through secondary ion mass spectroscopy (SIMS). There are now other applications of FIB technology that are being developed.

### 5.2. Proton Beam Writing

The Centre for Ion Beam Applications (CIBA) at the National University of Singapore is responsible for the development of a cutting-edge nano-lithographic technique known as proton beam writing (PBW), which is a technology that allows for direct writing in three dimensions. The use of accelerated protons (in the energy range of MeV), rather than electrons (in the energy range of keV), is a key aspect of PBW. Protons can penetrate deeper into materials like PMMA or SU-8 with a more defined path, making them a great choice for creating high-resolution, 3D microstructures as opposed to electrons in this approach [139].

In a single process, PBW can create well-defined structures with smooth and vertical walls, which is crucial for maintaining dimensional accuracy in high-resolution fabrication. PBW can produce features with a high aspect ratio and with dimensions smaller than 100 nm. This makes it a promising method for fabricating ultra-fine, complex structures. The penetration depth of the proton beam in the resist material (like PMMA) is dependent on the energy of the protons. A 1 MeV proton penetrates 20 µm deep, while a 3.5 MeV proton can go much deeper, up to 160 µm. This gives flexibility in controlling the depth of the exposure, which is important when creating structures with different depths or for writing multilayer devices [140].

The large mass difference between protons and electrons plays a significant role in why protons travel relatively undisturbed compared to lighter particles like electrons. Since the proton is much more massive, it retains most of its momentum during collisions with electrons, which results in minimal deflection or change in the proton’s trajectory. The concept of electronic stopping is key here—it refers to the energy loss due to collisions between the proton and the electrons in the material. However, because the proton has so much more mass and momentum, these interactions do not result in major changes to its path. Despite radiation damage causing secondary electrons to be produced, these electrons stay near the proton beam’s axis, leading to a less pronounced proximity effect than in electron-based lithography [141].

This property of protons is one reason why proton beams are commonly used in proton therapy for cancer treatment. Their ability to deliver high doses of energy with minimal scattering allows for precise targeting of tumors while minimizing damage to surrounding healthy tissue. Using 2 MeV proton beam nickel pillars of 400 nm and 5 µm have been successfully fabricated using PMMA as a resist [142]. It has been established that 3D nickel stamps may be created by utilizing PBW writing in conjunction with nickel electroplating. These stamps feature a nickel wall that is 100 nm broad and 2 µm high, which corresponds to an aspect ratio of 20. Manufacturing of high-aspect-ratio silicon needles with nanometer-sized tips has been accomplished with the use of PBW [143]. High-aspect-ratio test structures have been fabricated using PBW in SU-8 negative resist, demonstrating features of 60 nm with a depth of 10 µm [130].

**Figure 13:**
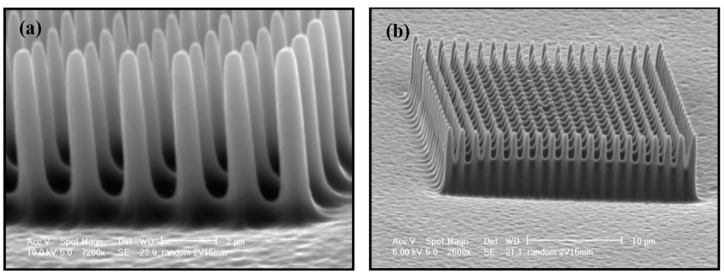
Arrays of silicon pillars with (**a**) 2 µm and (**b**) 1.2 µm periodicity fabricated by PBW. Copyright 2006 IAEA. Reproduced with permission from [145].

Metallic stamps of aspect ratio 20:1 (100 nm wide and 2 µm deep) were successfully fabricated in CIBA using PBW [139]. A high-brightness nano-aperture electron impact gas ion source (NAIS) which can generate ions with high spatial resolution from a small virtual source of 100 nm in an ionization chamber was experimentally examined by Liu et.al. [144]. Figure 13 [145] shows arrays of silicon pillars with a periodicity of 2 µm and 1.2 µm, respectively. It was possible to generate an axial Ar ion beam current of approximately 50 pA by employing an angular beam current density of 6.6 × 10^5^ A/Sr on the beam. According to the report, the greatest brightness was measured to be 750 Am^−2^ Sr, while the ion source energy spread was around 1 eV. Sub-10 nm H^2+^/H^+^ ion lithography is becoming increasingly possible with the present PBW; however, it is restricted by the low RF ion source brightness [144].

Nanoimprinting, silicon machining, medicinal applications, and photonics are some of the areas in which PBW is utilized, highlighting its potential for high-precision patterning in advanced manufacturing processes. PBW stands out due to its ability to create high-aspect-ratio structures with minimal proximity effects, which is a common challenge with techniques like electron beam lithography (EBL). Its use as an etch stop for creating porous silicon and its potential in biosensor and photonic device fabrication (like waveguides and microlens arrays) are also exciting, as these applications demand extreme precision at very small scales. The fact that PBW is still relatively new, with limited commercial instruments available, suggests there is a lot of room for future development. The possibility of reducing proton spot diameters to under 30 nm opens up exciting opportunities for 3D direct writing, potentially revolutionizing the fabrication of intricate micro- and nanoscale device [140].

### 5.3. Ion Projection Lithography

Ion projection lithography (IPL) offers a promising alternative or complement to current lithography techniques used in semiconductor manufacturing. By using ions such as protons, H_2_^+^, He^+^, and Ar^+^, IPL allows for finer patterning and the potential to continue scaling down feature sizes on chips, especially as traditional photolithography approaches its resolution limits. One of the main advantages of using ions instead of light photons in IPL is the precision with which the ions interact with the resist material. The light ions, due to their lower forward-scattering, can deliver a more defined dose to the resist without significant diffusion [146]. This means the printed features are more precise, and there is less risk of distortion, which is crucial when working at extremely small scales.

**Figure 14:**
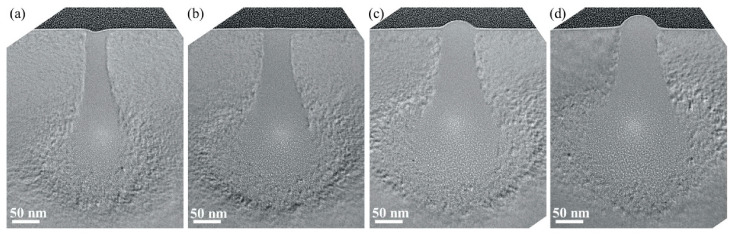
TEM images of silicon substrate cross-sections treated with He ion beam energy of 25 keV and ion doses of (**a**) 0.02 nC/µm; (**b**) 0.03 nC/µm; (**c**) 0.04 nC/µm; (**d**) 0.05 nC/µm. Copyright 2020 MDPI. Reproduced with permission from [147].

Additionally, IPL can operate in the 50 to 150 keV energy range, which is suitable for achieving high resolution. The ability to project these ions through a mask pattern and onto a substrate offers advantages in terms of depth of focus and stability in producing fine details, especially when compared to traditional optical lithography, which suffers from diffraction limits. The technique’s ability to work with medium-energy ions also means there is less energy transferred to secondary electrons, which could otherwise cause unwanted effects like proximity bias or pattern distortions. These properties make IPL particularly suited for producing advanced semiconductor devices with small geometries while maintaining high fidelity and low defect rates. Figure 14 [147] shows TEM images of cross-sections of silicon substrates exposed with He ion beam energy of 25 keV and various doses. A study by Melngailis et al. [128] demonstrates patterning down to 70 nm line-space pairs, along with innovations like the pattern lock servo system to address drift. Additionally, field distortion of less than 0.15 µm over 8 × 8 mm is measured, which agrees with the calculations.

On the basis of these accomplishments, a new generation of ion lithography machine has been built. This machine utilizes three times the demagnification and exposes a field measuring 20 mm by 20 mm with minimum dimensions of 0.12 µm. Furthermore, the ion optics introduce less than 10 nm of distortion [148]. These systems have demonstrated a significant number of the characteristics that are necessary for high-throughput lithography. With a single beam exposure, the ability to achieve a resolution of 50 nm has been demonstrated in certain areas of the exposure field and 75 nm across the entire field, which is 12.5 × 12.5 mm^2^ [149].

To perform ion projection lithography (IPL), it is necessary to have an ion source that possesses beam properties that are adequate. A small virtual source size (<10 µm) with a high emission current density (>0.5 A/cm^2^) and a finite energy spread (~1–2 eV) is the ideal ion source for intense pulsed light (IPL) [150,151]. This characteristic is referred to as the figure of merit. IPL systems use volume-plasma sources, and this process can be carried out with or without demagnification. In ion optical systems, chromatic aberration arises when ions with different energies do not focus on the same point in space. This means that ions with slightly different velocities have different focal points, leading to a blurred or distorted pattern on the substrate. This energy spread can distort the focused image, making it less sharp and precise, which is a significant problem when aiming for high-resolution lithography such as in IPL.

The multicusp volume-plasma source has largely replaced the duoplasmatron volume source in ion-projection printing because it produces a more stable and uniform ion beam, which helps reduce chromatic aberration and improve pattern resolution [152]. There are currently two IPL systems that are utilizing coaxial multicusp ion sources. One of these systems is known as the ALG-1000, a key IPL system developed by the Advanced Lithography Group (ALG), a consortium that includes industrial and research institutes from both the United States and Europe. Process Development Tool (PDT) is another IPL system that incorporates the coaxial multicusp ion source and is part of the MEDEA program, an international IPL development initiative [153]. These systems are part of the broader push to explore IPL as a potential solution for high-resolution patterning in semiconductor manufacturing, especially as feature sizes continue to shrink below the limits of traditional optical lithography.

### 5.4. Ion Beam Lithography Resists

The best formed features in ion exposed resist are typically found in PMMA, which is a positive tone organic resist. This is like the situation that occurs in X-ray or EBL applications. It is particularly effective for patterning in helium ion beam lithography (HIBL) because of its affordability, adhesion to silicon substrates, and good resolution capabilities. At the end of the 1970s, polymethyl methacrylate (PMMA) with an average molecular weight of 1.85 × 105 g per mole was patterned to a feature size of 2.7 μm using a helium ion dose ~17 μC/cm^2^ [154]. Shi et al. [155] explored the performance of higher molecular variants of PMMA for finer features utilizing both EBL and HIBL.

High contrast and higher etch resistance are two of the improved features that inorganic resists possess, in contrast to organic resists. HSQ (hydrogen silsesquioxane) is an inorganic negative-tone resist that has become an important material in high-resolution patterning, particularly for HIBL. HSQ is often diluted with a solvent like methyl isobutyl ketone (MIBK) to achieve the desired consistency and film thickness. Dilutions of 1:10 are used for thinner films (~5 nm), while a 1:1 dilution is used for thicker films (55–70 nm). Studies show that dot patterns were successfully created in HSQ films that were either 5 nm or 55 nm thick and the pitch of the patterns was 98 nm. For the 5 nm thick films, the dot width was around 6 nm, and for the 55 nm thick films, the dot width was around 14 nm. As a result of these findings, it was revealed that the HSQ resist exhibits a sensitivity that is approximately 4.4 times greater for helium ions (Ds~31 µC/cm^2^) compared to electrons (Ds ~137 µC/cm^2^), but the contrast values are nearly identical, approximately 2.1, for both forms of exposure. Helium ions have a higher yield of secondary electrons inside the thin resist compared to electrons of the same energy, which could enhance the exposure process and explain the greater sensitivity of HSQ in HIBL compared to EBL [156].

Additionally, Liu et al. [119] carried out lithographic patterning on hafnium-based inorganic resists. HafSO_x_ (a hafnium-based resist), like HSQ, is another advanced material used for high-resolution patterning, particularly in HIBL. HafSO_x_ shows a significant difference in sensitivity between helium ion exposure and electron beam exposure [157]. For helium ions, the required exposure dose is only about 4 µC/cm^2^ whereas for electrons, a much higher dose of 420 µC/cm^2^ is needed. This difference is likely due to the higher yield of secondary electrons generated by helium ions, which enhances the material’s sensitivity to ion beams.

The alumina-based resist is yet another type of inorganic resist that, when subjected to a helium ion beam, produces isolated lines of 5 nm in width and 20 nm in pitch. By using a 30 keV Focused Helium Ion Beam (FHIB), researchers have been able to control different pitch sizes, with pitches of 20 nm, 40 nm, and 64 nm being established. The dosages ranged from 200 to 700 µC/cm^2^, and the pitches were established. The lifespan of the resist solution, on the other hand, causes the resist to have some limitations. Cattoni et al. [158] demonstrated the fabrication of fine features (<10 nm) with alumina-based resist using HIBL.

For nanofabrication, a wide variety of chemically amplified resist compounds are being utilized in conjunction with various lithography techniques. Both chemically amplified resists (CARs) and nonchemically amplified resists (n-CAR) have been taken into consideration for use in HIBL operations. Numerous unique hybrids of organic and inorganic n-carbohydrates are now being produced to achieve this goal. According to this point of view, a new n-CAR MAPDSA-co-MAPDST hybrid resist for HIBL is a promising step forward in nanofabrication, particularly for achieving sub-20 nm features. Its hybrid nature and high sensitivity to helium ions offer significant advantages in terms of resolution and efficiency compared to traditional resists. The successful patterning of features as small as 20 nm at 60 µC/cm^2^ marks a key milestone, and this resist could potentially become an important tool for advanced semiconductor fabrication, nanotechnology, and emerging technologies. This resist exhibited an extremely low sensitivity of 7.2 μC/cm^2^ and a line edge roughness (LER) of 1.27 ± 0.31 nm [159].

One of the principal resists that were examined during the previous studies of proton beam writing (PBW) at CIBA was polymethyl methacrylate (PMMA). As a result of these investigations, the better patterning capabilities of PBW on PMMA, which are below 100 nm, have become widely distributed. According to the findings, trench widths of 65 nm and walls as narrow as 50 nm can be patterned in PMMA. CIBA has also been successful in achieving superior outcomes for the nickel electroplating process by using main PMMA molds that have been patterned with proton beams. Three-dimensional stamps with 100 nm features were fabricated for pattern transfer into PMMA films. These stamps consist of two raised platforms connected by several high-aspect-ratio ridges. The ridges are 100 nm wide, 2 μm deep, and 30 μm long [160]. The stamps are used to create test patterns by transferring them into PMMA, and this process involves physical imprinting of the features, which could potentially be combined with beam writing methods like PBW.

Sakai et al. [161] worked with a 75 µm thick acrylic film and optimized the fluence (the ion dose) for PBW. The optimal fluence for a 3 MeV proton beam was found to be approximately 9.0 × 10^13^ ions/cm^2^. The fluence is an important parameter in PBW because it dictates how many protons interact with the resist material, which in turn influences the resolution and quality of the patterned features. The fluence also needs to be carefully controlled to achieve the desired pattern resolution without overexposing or damaging the resist. Bolhius et al. [162] investigated the use of GG-developer, a commonly used developer in the LIGA process. They found that the GG-developer was capable of developing proton beam written structures in PMMA, with feature sizes down to 133 nm in a 2.4 µm thick PMMA layer. The GG-developer is specifically optimized to develop high-resolution patterns in thick PMMA films, making it well suited for PBW processes where deep patterns with fine features need to be produced.

Cutroneo et al. [163] worked on producing micro-channels in PMMA resist using PBW with ion fluences ranging from 3.1 × 10^13^ ions/cm^2^ to 3.7 × 10^14^ ions/cm^2^. The variation in fluence helps optimize the resolution and depth of the channels created in PMMA, showcasing the versatility of PBW in terms of exposure control and patterning capability. Erps et al. [164] reported the fabrication of SU-8 micro-pillars with a diameter of 20 µm and a height of 96 µm, which results in an aspect ratio of almost 1:5. Van Kan et al. [165] fabricated structures of 1.5 µm width with an aspect ratio of 24 using negative tone SU-8 resist and fabricated various other 3D structures with different proton energies.

HSQ is highlighted as another high-resolution negative resist for PBW. HSQ is known for its excellent resolution and high sensitivity to ion beams, making it ideal for sub-20 nm feature sizes. In one study, 20 nm wide structures were fabricated in 20 nm thick HSQ using 10 keV H_3_^+^ ions at a fluence of 7.5 × 10^13^ ions/cm^2^ [160,166]. For HSQ resist in thicker layers, 22 nm and 19 nm wide structures were achieved in 850 nm and 100 nm thick HSQ, respectively, using 1 MeV proton ions [160]. These results further show that PBW with HSQ can achieve sub-20 nm resolution even in relatively thick resist layers, which is important for multilayer patterning or when the resist needs to withstand multiple processing steps. Various other negative tone resists like ma-N, ma-P, AR-P 3200 series, TADEP, and KMPR have been studied with different proton beam energies and proton fluences to fabricate high-aspect-ratio 3D structures. Table 3 shows different types of resists used in ion beam lithography.

### 5.5. Advantages and Limitations

The fact that ions experience very little or no lateral scattering is one of the most significant advantages of ion beam lithography in comparison to electron beam lithography. Hence, the proximity effect in IBL is quite little. In the field of IBL, where the de-Broglie wavelength of a 150 keV H^+^ ion is approximately 10^−4^ nm, there are diffraction effects that are essentially non-existing. For diffraction, the depth of focus is approximately one millimeter. The IBL system is equipped with electrostatic lenses that can be tuned and have linear focal characteristics. These lenses also can electronically modify magnification. As a result of the low expected total system cost and the high throughput of 50–120 wafers per hour on 200 mm wafers, the tool operating cost is particularly appealing [127].

However, the presence of charged particles presents a hurdle for IBL systems, since it often results in pattern blur and inaccurate pattern placement. Because some ions can transmit through the resist and impinge on the substrate, ion beams can damage the substrates that lie beneath them if the energy is not effectively controlled. Additional research and development are required for the silicon membrane stencil mask technology that is utilized for IPL. The employment of stencil mask technology allows for the avoidance of issues involving ion scattering in transmission masks; however, it necessitates the creation of intricate patterns, which in turn necessitates the use of a secondary complimentary mask [169]. IBL is a promising technology with its own set of pros and cons, and other nano-lithographic techniques like nanoimprint lithography are developed.

## 6. Nanoimprint Lithography

A high-resolution parallel patterning method with cheap costs and great throughput, nanoimprinting lithography (NIL) relies on the naive idea of imprinting a pattern of nanoscale characteristics onto a substrate from a mold. This method was suggested by Stephen Chou et al. [170]. Initially, by replicating sub-25 nm vias and trenches to a depth of 100 nm, NIL demonstrated its great potential and fulfilled its promise of high-throughput patterning of nanostructures. Nanoimprint lithography (NIL) is a precision manufacturing technique used to create nanoscale patterns on substrates. At its core, the NIL process requires four essential components: a substrate, an imprint polymer (also known as resist), an imprint mold (or stamp), and an impression mask. The process begins by pressing the imprint mold, which contains the desired pattern, into the imprint polymer. This direct mechanical contact between the mold and polymer results in the transfer of patterns from the mold to the polymer, maintaining a one-to-one ratio between the mold features and the substrate [171,172].

During this imprinting process, a thin residual fluid layer is intentionally left behind between the mold and the substrate. This residual layer plays a critical role by preventing direct contact between the mold’s relief features and the substrate surface, acting as a buffer to reduce the risk of damage or collisions. The presence of this fluid layer ensures that the mold’s intricate patterns are transferred without distortion or impact. After the imprinting step, the imprint polymer undergoes a curing or hardening process either by photocuring or thermal curing depending on the type of polymer used [173]. Once the polymer is solidified, the mold can be removed, leaving behind the desired pattern imprinted on the polymer surface. To complete the pattern transfer, the residual layer is removed using an etching process, often an anisotropic plasma etch. This etching technique effectively strips away the remaining polymer layer, leaving the final, imprinted pattern transferred to the underlying substrate. The result is a highly detailed and accurate nanoscale pattern that can be used for a wide range of applications, such as semiconductor fabrication, photonics, and biotechnology [174]. Thermal NIL, ultraviolet NIL, and roll-to-roll NIL are three common techniques of NIL.

**Figure 15:**
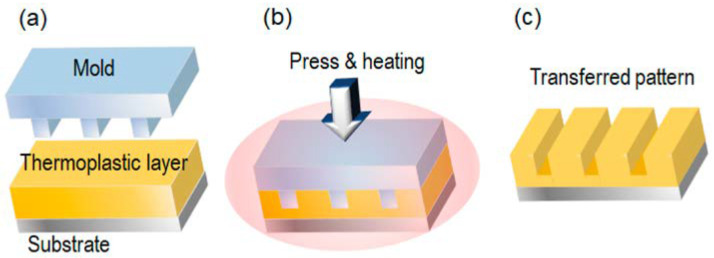
Thermal nanoimprint lithography process with a planar mold depicting (**a**) alignment; (**b**) pressing and heating; (**c**) pattern transfer and mold release. Copyright 2023 MDPI. Reproduced with permission from [175].

Thermal NIL is a high-resolution, cost-effective technique used to transfer nanoscale patterns onto a thermoplastic polymer (resist) on a substrate using heat and mechanical pressure. The process begins by applying a thin layer of resist, typically made from materials like Polymethyl Methacrylate (PMMA) or Polycarbonate, onto a substrate such as silicon or glass [175]. The resist is heated above its glass transition temperature (T_g_) to soften it, allowing it to conform to the mold’s pattern under pressure. After imprinting, the system is cooled, and the resist solidifies, locking the pattern in place. Any residual layer of polymer is removed through etching techniques like plasma or chemical etching to clean the final imprinted pattern. Thermal NIL is ideal for creating high-precision patterns, particularly for applications in semiconductor manufacturing, optics, and biotechnology [176]. The use of a planar mold in a thermal NIL process is illustrated in Figure 15 [175]. Thermal NIL has evolved to achieve increasingly smaller patterns, from 25 nm dot patterns to 6 nm, and ultimately 10 nm dots with a 40 nm pitch on PMMMA [177]. Despite challenges such as mold wear and the need for careful thermal and alignment control, thermal NIL offers significant advantages in terms of resolution, throughput, and material flexibility. As the technology continues to advance, it holds immense potential for the future of nanoscale fabrication and high-performance device production.

UV nanoimprint lithography (UV NIL) is a variant of NIL that uses ultraviolet (UV) light to cure the resist material instead of relying on heat, as in thermal NIL. It is a high-resolution, cost-effective lithographic technique used for fabricating nanoscale patterns on substrates, particularly for semiconductor, photonics, and biosensing applications [178]. The process starts with preparing the substrate, which can be made of materials like silicon, glass, or flexible substrates depending on the application. The substrate is coated with a thin layer of UV-sensitive resist material. The resist used in UV NIL is typically a UV-curable polymer that undergoes crosslinking when exposed to UV light. Common resists include UV-sensitive acrylates, epoxy-based resists, or PMMA doped with photosensitive components [179]. The resist is applied using spin coating or other techniques to ensure a uniform and thin layer. The mold used in UV NIL contains the nanoscale pattern to be transferred onto the substrate.

The mold is made from materials like silicon, quartz, or nickel, and it has finely patterned features that can range from several nanometers to microns in size. Once the mold is in contact with the resist, UV light is passed through the mold to expose the resist. The UV light causes the resist to undergo photopolymerization, curing and crosslinking the exposed regions [180]. This process solidifies the resist, locking the pattern into place. UV light is typically applied for a few seconds to a minute, depending on the resist material and light intensity. After the resist is cured, the mold is carefully removed [181]. Due to the solidified nature of the resist, the patterned structure is retained on the substrate. The mold removal is crucial to avoid damaging the newly imprinted features. The cured resist layer now serves as a mask for subsequent etching steps. Techniques like plasma etching or wet chemical etching can be used to transfer the pattern from the resist onto the substrate material, such as silicon or metal [182]. A 3D UV-curable NIL was demonstrated by Mohamed et al. [183], and perfusable channel array in a sub-micrometer to micrometer scale was fabricated by integrating NIL and UV lithography by Isobe et al. [184].

Roll-to-roll nanoimprint lithography (R2R NIL) is an advanced nanofabrication technique that combines the principles of NIL with continuous manufacturing processes. R2R NIL employs a continuous process that involves a flexible substrate (such as plastic film or foil) that is unwound from a roll, imprinted with nanoscale patterns using a mold, and then rewound after processing [185]. This system allows for high-volume, cost-effective production, which is a significant advantage over traditional photolithography techniques. R2R NIL is particularly attractive for applications that require large-area patterning on flexible or lightweight substrates [186]. The substrate, typically a flexible film (such as polyethylene terephthalate (PET), polyimide, or metal foils), is unwound from a roll. This substrate is then cleaned and coated with a thin layer of resist material, which is a polymer sensitive to the imprinting process [187]. This can be a thermoplastic polymer (used in thermal NIL) or a UV-curable polymer (used in UV NIL). A uniform layer of resist is applied to the flexible substrate, often using spin coating, doctor blade coating, or slot-die coating techniques. The application process is crucial to ensuring uniformity of the resist layer across the entire substrate.

Once the resist is applied, the continuous roll of substrate is passed through an imprinting station. The substrate is brought into contact with a mold that contains nanoscale features. The mold, typically made of silicon, quartz, or metal alloys, carries the pattern that needs to be transferred onto the substrate. In the imprinting process, pressure is applied to ensure that the softened resist fills the mold’s nanoscale features [188]. The imprint method can either be thermal NIL (where the resist is heated above its glass transition temperature) or UV NIL (where the resist is cured using UV light). After the imprinting process, the resist is either cooled (for thermal NIL) or exposed to UV light (for UV NIL) to solidify the resist, locking in the nanoscale pattern [189].

Once the resist is hardened, the mold is carefully removed, leaving behind the imprinted pattern on the substrate. The substrate moves continuously through the system, where each roll of material is imprinted with the nanoscale pattern and then rewound after processing. This allows for high-throughput production of large areas with a uniform pattern [190]. After the imprinting and curing steps, additional processing (such as etching, pattern transfer, or film deposition) may be performed to finalize the pattern and transfer it into the underlying substrate. Groh et al. developed an in-line metrology system for the patterning of 3D holographic structures using R2R NIL [191].

### 6.1. Nanoimprint Lithography Resolution

Nanoimprint lithography (NIL) has emerged as a powerful technique for high-resolution nanofabrication, demonstrating significant advancements in recent decades [192]. Chou et al. [193] achieved sub-10 nm resolution, imprinting arrays of 10 nm diameter holes with a 40 nm period in polymethylmethacrylate (PMMA), with the smallest feature reaching 6 nm. They successfully transferred these patterns to metal using liftoff and fabricated silicon quantum devices and nano-compact disks with a data density of 400 Gbits/in^2^, showcasing NIL’s potential for nano-device fabrication. Austin et al. [194] further advanced NIL by achieving 5 nm linewidth and 14 nm line pitch at room temperature and low pressure, demonstrating high uniformity over a 4-inch wafer and highlighting NIL as a cost-effective, high-throughput alternative to electron beam lithography for sub-35 nm applications. Recent studies have extended NIL’s resolution limits to sub-10 nm features using molecular dynamics simulations of polymer crosslinking to address shape retention challenges. Strategies such as optimizing resist formulations and introducing sacrificial bridge structures have shown promise in achieving sub-15 nm half-pitch structures with improved shape retention [195]. These developments underscore NIL’s potential for scalable, precise, and cost-efficient nanofabrication, making it a key technology for next-generation semiconductor devices and memory applications.

### 6.2. Advancement of New Materials in Nanoimprint Lithography

Nanoimprint lithography (NIL) is a cost-effective and high-throughput technique for creating polymer nanostructures. This method relies on the mechanical deformation of resist materials, which allows for resolutions beyond the limitations of traditional lithography [196]. The advancements in NIL depend heavily on the materials used for both the imprinting mold and the resist. Molds typically require high strength and durability, while resists need to be easily deformable, possess good mold-releasing properties, and exhibit sufficient mechanical strength [197]. Recent material developments include siloxane copolymers, which offer excellent mold-releasing properties, fast thermally curable liquid resists based on PDMS for rapid crosslinking, and UV-curable liquid resists that enable room-temperature nanoimprinting, expanding the applicability and efficiency of NIL [197,198].

Advancements in NIL have been propelled by crosslinked polymers, offering advantages over traditional thermoplastics like PMMA. These polymers, including thermally crosslinked mr-I 9000 and photochemically crosslinked mr-L 6000, can be processed from organic solvents, similar to thermoplastics, but achieve high thermal and mechanical stability through polymerization. Thermally crosslinked polymers undergo crosslinking during imprinting, dependent on the polymerization rate, while photochemically crosslinked polymers allow for lower imprint temperatures and shorter times. The use of these polymers has enabled the successful imprinting of structures with resolutions down to 50 nm, demonstrating their potential for creating patterns for etch masks and permanent applications [199]. Cedeno et al. [200] demonstrated the use of NIL for patterning organic semiconductors directly. Addressing mold release and pattern transfer challenges, siloxane copolymers, such as poly (dimethyl siloxane)-block-polystyrene (PDMS-b-PS) and related compounds, leverage PDMS properties like low surface energy and high silicon content, enhancing etching resistance in oxygen plasmas for high-resolution, high-aspect-ratio nanostructures [201].

Further progress relies on materials science, developing novel polymers and resins with optimized viscosity, crosslinking density, and surface energy to improve pattern transfer and reduce defects, alongside surface modification techniques and new template materials with enhanced release [202]. Recent efforts focus on liquid precursors curable at room temperature via UV light or heating, with UV-curable epoxysilicone materials undergoing cationic crosslinking, offering oxygen insensitivity, low shrinkage, and good dry-etching resistance, demonstrating successful patterning of features down to 20 nm at low pressures [198].

**Figure 16:**
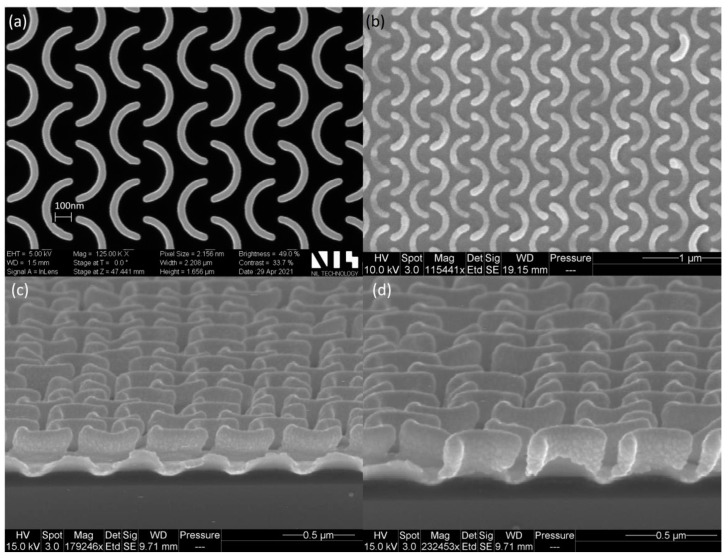
SEM images of (**a**) top view of the Si master; (**b**) top view of the C-shaped SERS pattern fabricated with UV-NIL; (**c**,**d**) cross-sections of UV-NIL SERS substrate. Copyright 2023 MDPI. Reproduced with permission from [192].

**Figure 17:**
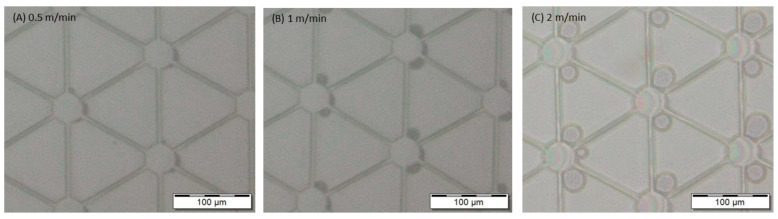
SEM images of a patterned surface after UV-NIL with different imprint speeds: (**A**) 0.5 m/min, (**B**) 1 m/min, (**C**) 2 m/min. Copyright 2021 MDPI. Reproduced with permission from [196].

Superplastic micro-forming of metallic glasses, such as Palladium-based amorphous alloy Pd_40_Cu_30_Ni_10_P_20_, has been explored, replicating nanometer-sized die shapes with high geometrical transferability, suggesting potential for mass production of nanodevices [203]. Metallic glasses, in general, offer superior mechanical properties for robust molds, capable of direct nanopatterning through hot embossing and crystallization for pattern replication [204]. NIL’s scope has expanded to encompass diverse materials like shape memory materials, covalent adaptive polymers, and porous materials, enabling applications in photovoltaics, water filtration, and smart materials, highlighting its growing impact [205]. Figure 16 [192] shows a C-shaped SERS nanopattern fabricated using UV-NIL and Figure 17 [196] demonstrates SEM images of a patterned surface after UV-NIL with different imprint speeds where the amount of air defects was found to increase on increasing the imprinting speed.

NIL is also being explored for organic electronics, utilizing materials like α-sexithiophene, poly(4-diphenyl-aminostyrene) (PDAS), and poly(phenyl-bis-4-aminostyrene) (PBAS) to modify thin film morphology, inducing smoother surfaces, and enabling patterned substrates with controlled optical and electrical properties, holding potential for advancing organic electronics, especially in FET fabrication. Metal-assisted chemical imprinting (Mac-Imprint) is a contact-based wet etching process that combines metal-assisted chemical etching (MACE) and NIL [206].

The Mac-Imprint method utilizes mesoporous metal catalysts to replicate silicon from polymeric molds, enhancing diffusion pathways and pattern transfer fidelity [207,208]. Gray-scale direct imprinting of porous substrates (DIPSs) enables three-dimensional patterning of porous nanomaterials, directly tailoring nanomaterial properties without intermediate resists, applicable in diffractive and plasmonic sensing [209]. These innovations aim to enhance NIL resolution, throughput, and reliability, expanding its applications across electronics, photonics, and biotechnology [206]. Table 4 displays the process conditions for various NIL materials.

### 6.3. Advantages and Limitations

Nanoimprint lithography (NIL) is a cutting-edge lithographic technique that overcomes the constraints of electron beam lithography and ultraviolet (EUV) lithography. It does not require any designed instruments and therefore is a lucrative technique that can manufacture high-resolution features. NIL is used to generate nanoscale patterns with high throughput, with a vast variety of use, along with simplicity and a high degree of adaptability, and it finds application in the production of LEDs and optoelectronic devices, solar cells, memory devices, and biosensors [221]. NIL, which is capable of replicating features with a size of less than 10 nm on wide area substrates, is a formidable candidate for enabling nanoscale manufacturing for the advancement of integrated circuits. The adhesion at the mold resist interface, on the other hand, raises the possibility of introducing damage to the nanopatterns; hence, the process of releasing the mold requires a considerable deal of care. In addition, the mold is prone to quick degradation due to substantial pressure and temperature that is applied between mold and substrate, which ultimately results in the mold needing to be replaced more frequently [222].

## 7. Alignment Marks in Lithography

Alignment marks are critical to precision and accuracy in nanoscale lithography. This ensures that the device navigates the correct layer layout and hence delivers appropriate device performance [223]. By aligning these marks, lithography tools compensate for distortions or changes in position caused by imperfections in the equipment, physical stress, or environmental factors [224]. Due to the size shrinking to the nano-level, slight deviations can cause significant deviations. This has led to the development of increasingly sophisticated alignment mark designs and detection methods tailored to various lithographic technologies, such as EUV lithography, EBL, and NIL [52].

Alignment markers in modern lithography have evolved from simple macroscale structures to complex fabrications that combine nanoscale features and high-contrast materials. For example, optical alignment systems use diffraction-based methods to detect the position of marks, while electron-beam systems directly image the mark structures with nanoscale precision. These marks also provide information about the quality of alignment. The system can be used to make real-time adjustments. Also, alignment marks not only serve as positional references but also offer data on alignment quality to quantify overlay errors [122]. Advanced systems use this feedback for real-time adjustments, improving lithography precision. Additionally, these marks are designed for compatibility with various substrates and conditions, enhancing their versatility across applications [225].

Additionally, improvements in materials used for alignment markers have greatly improved their durability and effectiveness [226]. High-contrast materials such as chromium and titanium nitride are now widely used to enhance marker visibility in various detection systems. These materials are resistant to chemical etching and heat treatment. This ensures that the markers last even in harsh production conditions. Emerging materials, including advanced polymers and nanocomposites, are also being investigated for their potential to improve alignment efficiency further [227].

### 7.1. Alignment Mark Designs

**Figure 18:**
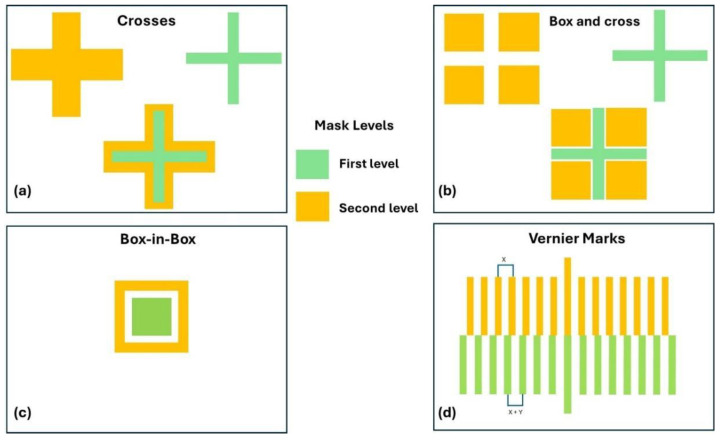
Schematic diagram of different alignment mark designs: (**a**) crosses; (**b**) crosses and boxes; (**c**) box in box; and (**d**) vernier marks.

The development of unique alignment marks suited to applications and technologies—such as basic cross-section points adequate for aligning base layers in lithography—has resulted from the necessity to manufacture a broad variety of instruments. Advanced technologies like extreme ultraviolet (EUV) lithography require more complex design for nanoscale precision. The type of alignment mark used also depends on the processing environment—marks must withstand conditions such as high temperatures or corrosive chemicals. Additionally, complex multilayer fabrication may use intricate marks like box-in-box designs to correct for translational and rotational misalignments. As lithographic techniques evolve, custom and application-specific marks tailored to unique process requirements and substrate characteristics become crucial, enabling precise alignment critical for modern, complex nanostructures. Figure 18 shows a schematic diagram of different types of alignment mark designs as in (a) crosses; (b) crosses and boxes; (c) box in box; and (d) vernier marks.

- **Crosses and Boxes**: The simplest and most used forms of alignment marks are crosses and dots. These marks are favored for their straightforward design and ease of use. Cross marks, consisting of two perpendicular lines, provide a clear intersection point that can be easily detected by alignment systems. Dots, on the other hand, are small points used as reference markers. Both types are used extensively in both lithography and electron beam lithography due to their simplicity and effectiveness in providing a clear, detectable reference for alignment purposes [228].
- **Box-in-Box Marks**: These marks are more complex and provide multiple reference points within a single mark. Typically, they consist of two concentric squares or rectangles, where the alignment is performed based on the position and orientation of the inner shape relative to the outer shape. This design allows for more detailed error analysis and correction, as it can provide information on both translational and rotational misalignments. These marks are widely used in multilayer processes where each layer’s exact positioning is crucial for the device’s functionality [229,230].
- **Segmented or Vernier Marks**: Named after the vernier scale used for precise measurements in mechanical engineering, these alignment marks are designed to measure misalignment more finely. They consist of two sets of lines where one set is slightly offset from the other. The degree of misalignment can be determined by observing the point at which the lines from the two sets appear to be continuous. This type of mark is extremely useful in fine-tuning the alignment process and is often used in conjunction with other types of marks to achieve high-precision overlays [231].
- **Grating Patterns**: For applications requiring higher precision, grating patterns are employed. These marks consist of a series of parallel lines or a grid, and they are particularly useful in advanced lithography techniques such as extreme ultraviolet (EUV) lithography. Grating patterns allow for the utilization of diffraction-based methods to precisely measure and correct alignment errors. The interference patterns generated by these gratings provide a more detailed feedback mechanism, enabling sub-nanometer precision in layer alignment. This type of mark is essential in applications where the overlay accuracy needs to be within a few nanometers [226,232].

### 7.2. Lithography Alignment Techniques

Lithography alignment techniques are pivotal in the semiconductor manufacturing process, ensuring the precise transfer of patterns from masks to wafers. These techniques are crucial for maintaining the integrity and functionality of the final semiconductor devices, and they vary significantly based on their operational principles and the technologies employed. Below is an in-depth look at the primary types of lithography alignment techniques utilized in the industry.

**Figure 19:**
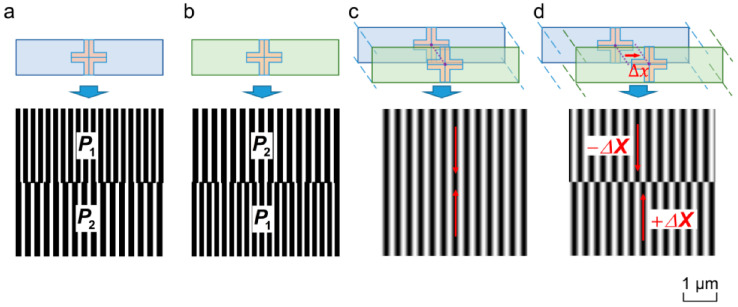
Grating pattern alignment marks for (**a**) wafer; (**b**) mask marks; (**c**) perfectly aligned fringe; (**d**) misaligned fringe. Copyright 2024 MDPI. Reproduced with permission from [232].

1. **Geometric Image Alignment**: This technique leverages the geometric properties of images projected during the lithography process. It involves aligning the patterns based on their shapes and relative positions as viewed through the lithographic optical system. The effectiveness of geometric image alignment is dependent on several factors, including the design of the alignment marks and potential distortions introduced by the optical lenses. These distortions can affect the precision of the alignment, making the quality of the optical components and the design of the alignment marks critical to the success of this technique [233].
2. **Light Intensity-Based Alignment**: This method utilizes variations in light intensity to facilitate precise alignment. It typically involves the detection of light reflected from specially designed alignment marks on the wafer, with the intensity of this reflected light compared against expected values to determine alignment accuracy. While straightforward, this technique can be susceptible to fluctuations in lighting conditions, which may impact its reliability. However, its simplicity makes it attractive for many standard lithographic applications where extreme precision is not the primary concern [234].
3. **Phase Shift Alignment**: This sophisticated technique uses phase-shifting masks to manipulate the phase of light waves passing through the mask. By creating points of destructive interference, phase shift alignment enhances the resolution of the patterning process, allowing to produce smaller features on the wafer. This method is particularly valuable in advanced lithography processes where reducing feature sizes is crucial for increasing the density and functionality of semiconductor devices [235].
4. **Moiré Fringe Alignment**: Figure 19 [232] shows grating pattern alignment marks for marginally distinct periods P2~1.1P1. Moiré fringe techniques employ the interference patterns that arise when two sets of gratings on the mask and the wafer overlap. These patterns are highly sensitive to misalignment, making them useful for achieving precise alignment with accuracies up to 10 nm and alignment ranges up to 500 µm. The technique involves a combination of coarse and fine alignment steps integrated into the same imaging process, allowing for both broad and highly precise adjustments. Moiré fringes can be generated using either physical or digital gratings, making this method versatile across different lithographic processes [232].
5. **Front-to-Back Alignment (FTBA)**: FTBA is essential for applications requiring the alignment of multiple layers, such as in the fabrication of microelectromechanical systems (MEMSs). This technique projects alignment marks from the back side of the wafer to its front side, ensuring precise layer-to-layer registration throughout the lithography process. Its ability to maintain high throughput makes it ideal for applications where speed and accuracy are both critical [236].
6. **Maskless Lithography Techniques**: These innovative methods, which include electron beam lithography and focused ion beam lithography, do not rely on traditional photomasks. Instead, they directly write patterns onto the wafer surface using beams of electrons or ions. While these techniques offer high resolution and are ideal for prototyping or manufacturing with low volume requirements, their throughput is generally lower than that of traditional masked lithography methods. This makes them less suitable for high-volume production but invaluable for custom or experimental applications where flexibility and precision are paramount [237].

### 7.3. Techniques for Implementing Alignment Marks

#### 7.3.1. Design Considerations

The design of alignment marks is critical in determining both the yield and performance of semiconductor devices. These marks must be carefully crafted to ensure they are detectable under various imaging conditions and resistant to processing steps that could degrade their visibility or geometric integrity. The size, shape, and materials used for alignment marks are selected based on their visibility in the chosen lithography system and their compatibility with the processing environment. For instance, smaller marks are less obtrusive and conserve valuable wafer space but may be harder to detect, especially under less-than-ideal imaging conditions. Conversely, larger or more complex marks can be easier to detect but consume more wafer real estate. The contrast between the marks and the substrate is also vital; high-contrast materials such as chromium or titanium nitride might be used to ensure marks are easily discernible against the silicon background. Moreover, the design must account for potential distortions due to lens aberrations, focus variations, and process variations such as etching or deposition inconsistencies. Marks must be robust enough to maintain their integrity through multiple layers of processing, as any degradation can lead to misalignment and thus lower the yield and performance of the final device [238,239].

#### 7.3.2. Placement Strategies

The placement of alignment marks on masks is another strategic consideration that can significantly influence the accuracy and effectiveness of the lithography process. Optimal placement strategies ensure that marks are accessible and readable by alignment systems throughout the fabrication process, even after various processing steps that might obscure or damage earlier marks. One common strategy is to place alignment marks at the corners of the exposure field as well as near the center. This arrangement allows for comprehensive coverage and ensures that any distortions or shifts in the wafer can be detected and corrected. It is crucial that the marks are placed away from areas of critical device structures to avoid interference but still close enough to areas of interest to provide effective alignment cues.

In multilayer processes, alignment marks may need to be replicated on each layer, or new marks may be added in subsequent layers to account for any interlayer misalignment. The alignment marks on each layer must be precisely aligned relative to those on previous layers to ensure accurate stacking of features. This can involve the use of interlayer alignment strategies, where marks are designed to interlock or align with marks on adjacent layers, enhancing the overall alignment accuracy. Advanced techniques, such as the use of “global” and “local” alignment marks, are also employed. Global marks are used for the initial rough alignment of the wafer, while local marks, placed closer to the areas of active device fabrication, are used for fine alignment within a specific region of the wafer. This tiered approach allows for high-precision alignment that is crucial for advanced device structures, particularly at nodes below 10 nm where even minor misalignments can be catastrophic. Furthermore, the alignment mark placement must consider the entire process flow, including any potential for mark degradation due to subsequent processing steps such as etching, chemical mechanical polishing, or high temperature anneals. The strategic placement and robust design of alignment marks are essential not only for maintaining alignment accuracy but also for maximizing the yield and performance of the fabricated devices [239].

## 8. Overview of Mask Fabrication

The mask making process is fundamental to lithography in semiconductor manufacturing, where masks serve as critical stencils for transferring circuit patterns, defining features at micro- and nanoscale levels. This highly precise process utilizes advanced materials and technologies, beginning with the design and layout of circuit patterns via computer-aided design (CAD) software. These patterns are then meticulously transferred onto mask substrates through lithography and etching. The accuracy of these masks is crucial, as any imperfections can significantly impact the yield and functionality of the final semiconductor products. Understanding the various types of photomasks and the materials used for their fabrication is vital due to their central role in lithography. This knowledge is essential for choosing suitable mask types for specific lithographic requirements and for driving the development of innovative mask technologies to address the evolving challenges of modern semiconductor design and production.

### 8.1. Photomask Types

Photomasks are essential tools in the lithography process for semiconductor manufacturing, functioning as precise templates that transfer circuit patterns onto wafers. The type of photomask chosen is critical as it directly influences the resolution and quality of the final semiconductor devices [240]. There are several types of photomasks, each designed to meet specific requirements of modern lithography systems:

- **Binary Masks**: These are the most straightforward type of masks, consisting of clear and opaque regions. The opaque areas block the light, while the clear areas allow light to pass through, directly transferring the pattern onto the wafer. Binary masks are widely used for their simplicity and effectiveness in less complex lithography techniques.
- **Phase-Shifting Masks (PSMs)**: Phase-shifting masks enhance the resolution and image quality by shifting the phase of light passing through the mask. This shift creates constructive and destructive interference patterns, sharply defining the edges of the features on the wafer. PSMs are particularly effective in advanced lithography, where feature sizes are close to or below the wavelength of the exposing light.
- **Attenuated Phase-Shifting Masks**: These are a variant of the traditional phase-shifting masks, incorporating a partially transparent film that reduces the intensity of the transmitted light. This attenuation helps in improving the process latitude and contrast of the image on the wafer, allowing for even finer resolution. These masks are beneficial in applications requiring extremely small feature sizes and high-density patterns [241].

Each type of photomask plays a specific role in semiconductor manufacturing, depending on the complexity of the design and the resolution requirements of the lithography process. The selection of the appropriate mask type is driven by factors such as the desired feature size, the optical properties of the lithography system, and the specific constraints of the semiconductor device being fabricated. As semiconductor technology continues to advance, the development and refinement of photomask types remain central to achieving higher resolutions and better device performance.

### 8.2. Materials Used in Photomask Fabrication

The choice of materials used in photomask fabrication is crucial, as it directly impacts the performance, durability, and quality of the masks used in semiconductor lithography. The materials must possess specific properties such as optical transparency, chemical resistance, and mechanical stability. Here are some of the primary materials employed in the construction of photomasks:

- **Quartz**: Quartz is the most commonly used substrate material for photomasks due to its excellent optical transparency in the ultraviolet (UV) range, which is crucial for lithography processes involving deep UV and extreme ultraviolet (EUV) light. Quartz also offers superior dimensional stability and resistance to thermal expansion, which are essential for maintaining pattern accuracy during the intense UV exposure in lithography processes. Its high chemical resistance ensures that it can withstand the rigorous cleaning and processing chemicals used during mask fabrication and wafer processing [242].
- **Chrome**: Chromium is used to form the opaque regions on photomasks. It is deposited on the quartz substrate to create the patterned features that define the mask’s circuit designs. Chrome’s high opacity to UV light makes it an ideal choice for creating sharp, well-defined lines that block light effectively. Additionally, chrome adheres strongly to quartz, providing durability and resistance to peeling or flaking during the mask usage. Its ability to be finely patterned with high precision through processes like electron beam lithography ensures that even the most intricate designs can be accurately rendered [243].
- **Titanium Nitride**: Used in advanced photomasks, titanium nitride (TiN) serves as an alternative to chromium for certain applications, particularly in phase-shifting masks. TiN can be used to form attenuated phase-shifting areas due to its semi-transparent properties at specific lithography wavelengths. This material is also known for its mechanical hardness and chemical stability, which are beneficial for the longevity and reuse of masks in high-volume production environments [244].
- **Molybdenum and Silicon thin films**: Another material used in advanced mask technologies, particularly for EUV lithography, is molybdenum and silicon thin films. It is chosen for its ability to withstand the high-energy photons used in EUV lithography without degrading. This material provides a low degree of absorption and thermal stability, which are crucial for maintaining mask integrity under the intense conditions of EUV exposure [245].

The selection of materials for photomask fabrication is guided by the specific requirements of the lithography process, including the exposure wavelength, feature size precision, and environmental durability. Advances in material science continue to play a pivotal role in the development of new photomask technologies that push the boundaries of what can be achieved in semiconductor manufacturing [246].

### 8.3. Challenges in Mask Fabrication

#### 8.3.1. Resolution Limits

In the realm of semiconductor manufacturing, the quest for miniaturization continues to push the limits of lithography and mask-making. As feature sizes decrease, achieving high resolution in mask-making becomes significantly more challenging. This drive towards smaller features confronts a fundamental barrier in optical lithography, where the resolution is constrained by the wavelength of light used for exposure. The resolution limit, often described by the Rayleigh criterion, delineates the smallest feature size that can be reliably resolved. In practical terms, as the industry moves towards node sizes below 10 nm, traditional optical lithography struggles to maintain fidelity, prompting a shift towards advanced techniques such as extreme ultraviolet (EUV) lithography. EUV uses significantly shorter wavelengths, allowing for finer patterning, but it also introduces complexities in mask fabrication. Mask features must now incorporate intricate, smaller-scale designs that are extremely challenging to produce and replicate accurately [247]. To push the boundaries of resolution, ongoing innovations in both materials and mask-making technologies are critical. This includes the development of new resist materials that can capture finer details and the use of sophisticated etching techniques that can accurately transfer these minute patterns onto the mask substrate. Additionally, advances in electron beam lithography, which can bypass some of the limitations of light-based lithography, offer another avenue for enhancing resolution in mask production [248,249].

#### 8.3.2. Defect Mitigation

Defects in mask-making, such as particulates, pinholes, or inaccuracies in pattern generation, pose significant challenges to the lithographic process. These defects can propagate through to the wafer, leading to malfunctioning circuits and reduced yield. The impact of such defects becomes exponentially more significant as feature sizes shrink, where even the smallest flaw can render a device non-functional [250]. To ensure the high quality and reliability of masks, several strategies and technologies are employed. Advanced inspection systems play a crucial role in this aspect. Techniques such as optical and electron microscopy are used to scrutinize masks at various stages of the fabrication process, detecting anomalies that could affect performance [251]. Once defects are identified, precise repair technologies are deployed. These may involve material deposition to fill pinholes or focused ion beams to correct pattern inaccuracies. Moreover, cleanroom environments are meticulously maintained to minimize the introduction of particulates during mask production [252]. Automation in mask handling and storage also reduces the risk of human-induced defects. In the broader scope, statistical process control and continuous improvement practices are integral to refining mask-making processes, thereby reducing the incidence of defects over time [253]. Overall, addressing these challenges involves a combination of cutting-edge technology, stringent process control, and ongoing innovation. These efforts are vital for sustaining the advancement of semiconductor technology, enabling the production of more powerful and reliable electronic devices.

## 9. Molecular Strategies in Nanoscale Lithography

As the quest for smaller, more powerful devices continues, advanced lithography techniques face increasingly stringent demands for resolution, precision, and throughput. Traditional lithographic materials and methods are reaching their fundamental limits, necessitating a paradigm shift towards molecular-level control and innovative material design. The incorporation of molecular strategies has become essential to overcoming these challenges and realizing the full potential of nanoscale fabrication [254].

This section focuses on the critical role of molecular materials in pushing the boundaries of lithography. We explore how the design and properties of these materials, engineered at the molecular level, directly influence the performance and capabilities of advanced lithographic processes. By harnessing the principles of molecular chemistry and materials science, researchers are developing novel resists, templates, and techniques that enable unprecedented precision in pattern transfer and device manufacturing.

Our discussion begins with an examination of resist materials, the cornerstone of lithography. We delve into the molecular composition, properties, and reaction mechanisms that govern their behavior during exposure and development. Furthermore, we explore nanostructured films, with an emphasis on self-assembling materials, and discuss how the molecular-level organization of these films can be leveraged to create intricate patterns and functional devices.

### 9.1. Molecular Materials for Lithography

#### 9.1.1. Resist Materials: Molecular Design and Properties

Molecular resists represent an alternative to traditional polymer resists in lithography, particularly for extreme ultraviolet (EUV) and electron beam lithography (EBL) [255]. These materials are designed with the goal of improving resolution and reducing line edge roughness (LER), which are critical challenges in next-generation lithographic techniques [256]. Molecular resists offer the potential for reduced pixel size and greater synthetic control, as they are composed of single molecules with precisely defined structures. The design of molecular resists often involves incorporating all the functionalities needed for a chemically amplified resist (CAR) into a single molecule. This can include a photoacid generator (PAG) moiety bonded to a protected polyphenol derivative. Having a single-component resist can ensure uniform PAG density and better control over acid diffusion, which influences both resolution and LER [257]. Wang et al. [258] synthesized and characterized a novel molecular glass (TPSiS) with a photoacid generator for use as a single-component molecular resist in electron beam lithography. The TPSiS resist demonstrates high resolution, resolving 25 nm dense line/space patterns, and exhibits potential for application due to its etching selectivity.

Y. Wang et al. [259] designed and synthesized molecular resists based on a bis(4-butoxyphenyl) sulfone core attached to a varying number of radiation-sensitive triphenylsulfonium units (BPSSn, where n = 2, 3, and 4). These materials were evaluated for their physical properties, including solubility, film-forming ability, and thermal stability, to assess their viability as photoresist materials. The BPSS4 resist demonstrated high resolution (16/13 nm) and low line edge roughness (2.5/2.5 nm) in e-beam and EUV dense line patterning, respectively. The BPSS4 resist also exhibited high etch resistance and accurate pattern transfer capabilities, making it suitable for high-resolution lithography applications. Kim et al. [260] synthesized a novel molecular resist material based on polyhedral oligomeric silsesquioxane with diazoketo groups for deep UV lithography. Initial lithographic evaluations suggest the new material platform’s potential for next-generation resists.

**Figure 20:**
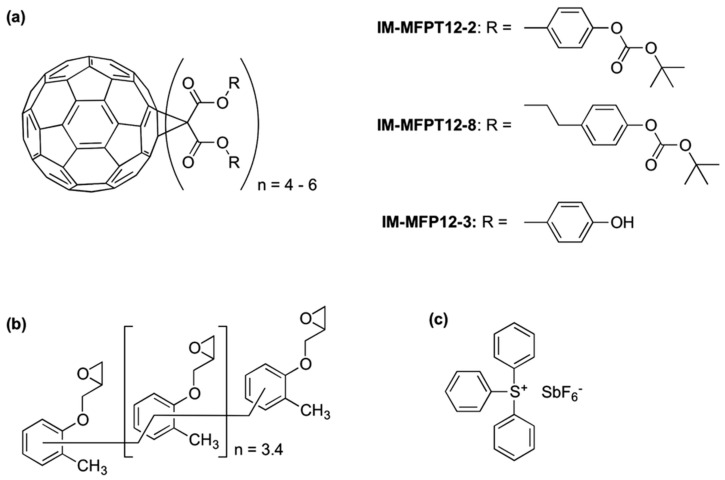
Molecular structure of phenol-based fullerene chemically amplified resists. (**a**) IM –MFP12 –2, IM –MFP12 –8, IM –MFP12 –3; (**b**) IM –MFP12 –2 and IM –MFP12 –8 combined with an epoxy crosslinker; (**c**) IM –MFP12 –2 and IM –MFP12 –8 combined with triphenyl sulfonium hexafluoro antimonate. Copyright 2014 Royal Society of Chemistry. Reproduced with permission from [262].

Molecular resists can be designed for both positive and negative tone imaging. In negative tone imaging, the BPSS materials allowed for negative patterning through organic development in both e-beam and EUV lithography [261]. In positive-tone resists, the material becomes soluble upon exposure and subsequent development, while negative-tone resists become insoluble. The development of molecular resists often involves optimizing the dissolution behavior to ensure high chemical contrast and reduced swelling during the development process. A new family of fullerene-based negative tone chemically amplified e-beam resists, using industry compatible solvents, has been developed. The molecular resist core is often synthesized through the acid-catalyzed condensation of phenol with a ketone or aldehyde and then functionalized [262]. The molecular structures of a new family of phenol-based fullerene derivatives are shown in Figure 20 [262].

The PhD thesis of Dr. Jedsada Manyam explores Next-Generation Lithography (NGL) technologies, particularly focusing on the development and optimization of chemically amplified fullerene-based resists for electron beam lithography [263]. It addresses the limitations of conventional polymeric resists and aims to achieve high resolution, sensitivity, and etch resistance for advanced microfabrication. A sensitivity of ~40 μC/cm^2^ was achieved at 20 keV. Isolated features with a line width of 13.6 nm as well as ~20 nm lines on a 36 nm pitch were patterned, whilst one variant demonstrated resolution to 15 nm half-pitch at slightly higher dose.

While early molecular resists have demonstrated promising results, including high resolution and low LER, challenges remain [264]. These include achieving sufficient sensitivity, preventing pattern collapse, and optimizing the development process [265]. Further research and development are focused on addressing these challenges and exploring the full potential of molecular resists for advanced lithography. Some approaches involve blending molecular resist components to adjust sensitivity. Molecular resists, including oligomers, molecular glasses, discotic liquid crystals, and inorganic materials, have garnered attention as promising candidates [266].

#### 9.1.2. Nanostructured Resists: Self-Assembling Materials for Advanced Patterning

Nanostructured resists leverage the self-assembly properties of materials to create patterns at the nanoscale, offering a route to high-resolution and high-throughput lithography. This approach moves beyond traditional resist materials and processes by utilizing the spontaneous organization of molecules or nanostructures into ordered arrays or templates [86]. One approach involves using structures generated by dip-pen nanolithography (DPN) as positive resists for fabricating nanohole arrays [267]. In this method, the DPN-generated nanostructures serve as a mask, protecting the underlying material during etching. Polymer pen lithography (PPL) is a cantilever-free scanning probe-based technique used for molecular printing that can generate sub-100 nm molecular features in a massively parallel fashion [268]. PPL enables the creation of combinatorial arrays of nanostructures composed of proteins and metals, making it suitable for high-throughput screening and biologically relevant applications.

Another avenue in nanostructured resists is the use of block copolymers (BCPs), which self-assemble into periodic nanostructures with domain sizes that can be controlled by the molecular weight and composition of the polymer [269]. These self-assembled nanostructures can be used directly as a resist or as a template for further pattern transfer. Beyond block copolymers, other self-assembling materials, such as liquid crystals and supramolecular assemblies, are being explored as potential nanostructured resists. These materials offer a diverse range of morphologies and functionalities, enabling the creation of complex patterns with nanoscale precision. Chang et al. [270] review self-assembled nanocomposites and nanostructures for environmental and energy applications, highlighting their use in wastewater purification, hydrogen production, energy storage, and energy harvesting. The review discusses the classification, synthesis, characterization, and properties of these materials, aiming to demonstrate the advantages of self-assembled nanostructures and provide perspectives for future research. Self-assembled nanomaterials offer improved ionic transport and electronic conductivities and can tolerate high currents, offering a solution for high-power energy storage.

E-beam lithography (EBL) is a powerful tool used alongside nanostructured resists for generating nanostructures and fabricating nanodevices with fine features approaching a few nanometers in size. EBL does not require a template, can easily write quickly, and can expose a thick resist without ion contamination [86]. While nanostructured resists hold significant promise, challenges remain in controlling the self-assembly process, achieving high pattern fidelity, and integrating these materials into existing lithographic workflows. However, the potential benefits in terms of resolution, throughput, and cost-effectiveness make nanostructured resists an active area of research and development for advanced lithography [53].

### 9.2. Molecular Mechanisms in Lithography

#### 9.2.1. Photoresist Chemistry: Photochemical Reactions and Processes

Photoresist chemistry is a critical factor in extreme ultraviolet (EUV) lithography, requiring the development of materials with high sensitivity to achieve efficient patterning at reduced feature sizes [271]. Out-of-Band (OoB) radiation, approximately 4% of the total radiation from EUV sources, can negatively impact resist performance [272,273]. Photoacid generators (PAGs) with selectivity to EUV radiation are designed and synthesized to minimize the effect of OoB by decreasing deep UV (DUV) absorption through incorporation of insensitive cations [224]. Both blended PAG and polymer-bound PAG resist formulations utilizing these PAGs have demonstrated improved EUV sensitivity [274].

Significant effort is focused on chemically amplified resists (CARs) for EUV lithography [48]. Studies show that metal salt sensitizers improve both EUV photon absorption and electron yield, resulting in higher sensitivity. For instance, the incorporation of a specific metal sensitizer with high EUV absorbance could improve sensitivity by 17% without significantly impacting LWR or local critical dimension uniformity (LCDU) [266]. Yamamoto et al. [56] improved sensitivity by 43% by adding a metal sensitizer. Metal sensitizers improve both EUV photon absorption and electron yield resulting in higher sensitivity [275]. Higher absorption of anions increases electron yield by merely higher absorption but has limited impacts on electron generation efficiency; however, cations of metal sensitizer dictate electron generation efficiency. Halogenated sensitizers, such as fluorine and iodine, also help with electron generation due to their higher absorption, but sensitivity depends heavily on the chemical environment of the halogen bonds [273]. However, higher electron yield does not always translate to higher sensitivity for all halogen sensitizers [276]. The chemical environment where these halogens are attached has a significant impact on the sensitivity.

Molecular CAR systems are also actively researched. Cyclic low-molecular-weight (CLM) resists have achieved sub-30 nm half-pitch resolution with high sensitivity [266]. Noria derivatives with pendant adalantyl ester groups have produced 25 nm resolution patterns using EUV lithography at doses less than 10 mJ/cm^2^ [277]. Modified Noria molecules with oxetane crosslinking moieties have been used in negative-tone CARs, achieving 20 nm resolution with 3.2 nm LER [278]. Molecular glass (MG) resists based on calixarene cores are also being explored as alternatives to polymeric resists, achieving 28 nm 1:1 space line using EUV exposure [279]. The multi-trigger resist concept, which involves regenerating photoacids, is being explored to achieve high sensitivity solubility changes above a certain dose threshold and sharper lines with lower LER [280].

**Figure 21:**
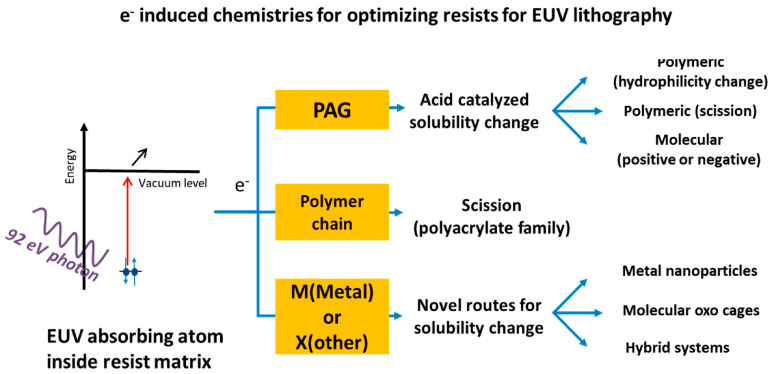
Schematic diagram of a summary of the evolution of the EUV resists optimized by electron-induced chemistry. Copyright 2020 MDPI. Reproduced with permission from [48].

Inorganic resists, including metal oxides, are also under investigation, with some hybrid materials showing 26 nm lines with a 4.2 mJ/cm^2^ EUV dose [281] and directly patternable metal oxide hardmasks achieving 13 nm half-pitch at 35 mJ/cm^2^ and 11 nm hp with 1.7 nm LWR [282]. Tin-oxo cages have been found to have dual tone property which showed positive tone behavior when irradiated with a low dose of EUV or E-beam and a negative tone behavior when it is irradiated at higher dosages [283]. Organohydrogensilsesquioxane-based EUV resists have shown excellent etch selectivity and ability to form patterns by using industry standard TMAH development process, as well as low LWR (<2 nm) with sufficient sensitivity (40–60 mJ/cm^2^) [284]. Figure 21 [48] shows a summary of the evolution of the EUV resists optimized by electron-induced chemistry.

#### 9.2.2. Molecular Dynamics Simulations: Predictive Modeling of Nanoscale Behavior

**Figure 22:**
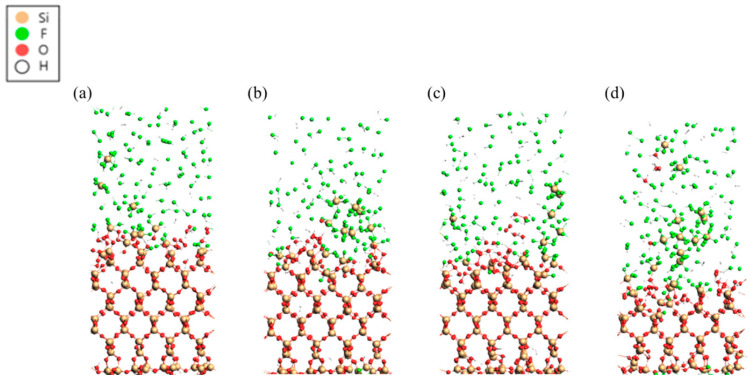
Simulation results of plasma etching of SiO_2_ with HF with different ion energies of (**a**) 20 eV, (**b**) 30 eV, (**c**) 40 eV, and (**d**) 80 eV. Copyright 2021 American Chemical Society. Reproduced with permission from [291].

Molecular dynamics (MD) simulations are indispensable for understanding and predicting material behavior at the nanoscale, providing insights into lithographic processes that are challenging to observe experimentally [285]. MD enables the modeling of atom and molecule dynamics, crucial for investigating phenomena such as resist material behavior, EUV-induced processes, etching, and self-assembly. Regarding EUV resists and lithography, simulations enable study of the following:

- **Resist Materials**: MD can illuminate how resist materials behave during lithographic steps such as exposure, development, and etching. For example, MD can provide insights into the dynamics of polymer chains during pattern formation and the interactions between resist components and solvents. Such insight may lead to improved resist formulations. A study by Kim et al. [286] used coarse-grained MD simulations to investigate the effect of chain conformation on line-edge roughness (LER) formation in EUV photoresists, revealing the importance of balancing chain length and polymer–developer interactions to minimize roughness [286,287]. This simulation of EUV photoresists is useful in designing a material and process for sub-1x nm patterning, as the critical dimension is almost the molecular size [288].
- **Nanoelectrode Lithography**: MD simulations can provide important insights into the underlying mechanisms of novel lithography techniques. Reactive Force Field Molecular Dynamics (ReaxFF MD) simulations have been applied to study nanoelectrode lithography (NEL) for the oxidation of silicon surfaces, including the effects of pulse duration and electric field strength on oxide formation [289].
- **Etch Processes**: MD simulations are employed to model plasma etching, simulating the interactions of ions and radicals with resist materials and substrates. This aids in optimizing etching conditions and designing etch-resistant materials, as demonstrated in studies using MD to analyze the effects of plasma composition and process parameters on etch selectivity and pattern transfer [290,291,292,293]. Figure 22 [291] shows a plasma etch simulation of SiO_2_ with HF with different ion energies of (a) 20 eV, (b) 30 eV, (c) 40 eV, and (d) 80 eV. Simulation results show that the amount of SiO_2_ etched increases with the increase in incident ion energy of each HF molecule.

- **Self-Assembly**: MD simulations are valuable for studying the self-assembly of nanostructures like block copolymers used in nanostructured resists. These simulations provide insights into the factors governing the morphology and ordering of self-assembled structures and molecular self-assembly of photoresist materials, aiding in the design of materials with tailored properties [294,295].
- **Photolithography Development Process**: MD can predict the behavior of surfactants in photoresist during development process [296]. Results found that the concentration of surfactant affected the surface structure, which could potentially affect the yield of manufactured devices [297,298].

MD simulations provide atomic-level details, making them a powerful tool for understanding lithography’s complex molecular mechanisms. The predictive capabilities of MD simulations can also accelerate the discovery and optimization of lithographic materials and processes, paving the way for advanced nanofabrication techniques. The increasing computational power and improved accuracy of force fields make MD simulations an essential component of modern lithography research, but they also present challenges. Overcoming these limitations will further enhance the role of MD in advancing nanofabrication [299,300].

### 9.3. Molecular Scale Patterning Techniques

#### 9.3.1. DNA Origami: Bottom–Up Fabrication of Complex Nanostructures

DNA origami is a bottom–up nanofabrication method that allows for the creation of arbitrary two- and three-dimensional shapes at the nanoscale. This technique utilizes the specificity of base pairing between complementary DNA strands to construct complex structures by folding a long single-stranded DNA molecule (often from a viral source) with the help of numerous shorter “staple” strands [301]. These staple strands bind to specific sequences on the scaffold strand, causing it to fold into a desired shape [302]. DNA origami serves as a molecular breadboard, integrating bottom–up (bio)chemistry with macroscopic devices created by top–down lithography [303]. Martynenko et al. [304] explored how DNA origami serves as a molecular breadboard, integrating bottom–up (bio)chemistry with top–down lithography at nanometer resolution. By using self-assembled colloidal nanoparticles instead of top–down patterning, the complexity of manufacturing is reduced, making the positioning of molecules and nanoscale objects on macroscopic arrays more accessible.

The DNA origami technique was developed by Paul Rothemund at the California Institute of Technology [305]. The process involves designing the base sequences of DNA to enable self-assembly into a variety of shapes. Computer-aided design (CAD) software, such as caDNAno, assists in designing the placement of staple strands, ensuring accurate folding and reducing errors. The precisely designed staple strand sequences are synthesized in a lab and then mixed with the scaffold strand in a buffer solution. Subjecting this mixture to a specific temperature cycle allows the staple strands to find and bind to their complementary sequences on the scaffold strand, causing it to fold into the desired shape through hydrogen bonding. Created designs can be observed through methods including electron microscopy, atomic force microscopy, or fluorescence microscopy [306]. Tørring et al. [307] highlighted DNA origami as a promising method for the spatially controlled positioning of functional materials via self-assembly, a central goal in nanotechnology. Their tutorial review discusses the basic design principles, organization of functional materials, and recent implementations in DNA robotics, along with future challenges and opportunities.

**Figure 23:**
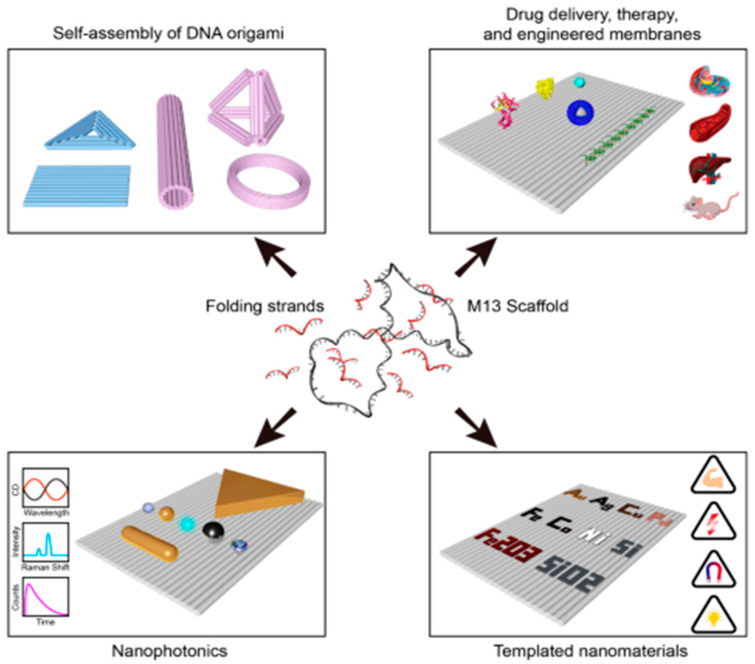
Schematic diagram of DNA origami applications and its engineered nanomaterials. Copyright 2023 American Chemical Society. Reproduced with permission from [306].

DNA origami structures can act as templates for nanoscale patterning, with applications in biotechnology [308]. They can be used to create plasmonic, entirely metallic nanostructures in a parallel manner on different substrates. DNA origami can also organize individual molecules and nanostructures with nanoscale spatial resolution and single-molecule control. Functionalized DNA origami can be selectively placed into nanoarrays using thermal scanning probe lithography (t-SPL), enabling the construction of hetero-functionalized biomolecular nanoarrays with applications in bionanotechnology and materials science [303]. Figure 23 [306] shows a schematic diagram of DNA origami engineered nanomaterials and various applications.

DNA origami leverages programmable self-assembly of DNA strands to create precise 2D/3D nanostructures, enabling bottom–up fabrication of sub-10 nm features [309]. This technique complements top–down lithography methods like nanoimprint lithography (NIL) by providing molecularly precise templates for patterning metallic nanowires or quantum dots [310,311]. For example, DNA origami scaffolds guide the deposition of lithographic resists, enhancing resolution in EUV lithography by reducing line-edge roughness through controlled nanoparticle placement [312]. Its ability to generate complex, customizable geometries make it ideal for prototyping nanoscale devices such as biochips while avoiding the high costs of traditional mask-based lithography.

#### 9.3.2. Biomolecular Templates: Guiding Assembly at the Molecular Level

Biomolecular templates harness the inherent self-assembly properties of biological molecules to direct the organization of materials at the nanoscale [313]. This bottom–up approach leverages the precise interactions between biomolecules like DNA, proteins, and viral particles to create defined arrangements, offering an alternative to top–down lithographic techniques. Molecular self-assembly is a process where molecules adopt a defined arrangement without external guidance, driven by non-covalent interactions such as hydrogen bonding, hydrophobic forces, and electrostatic interactions. Biomolecular assembly is a key process that drives structure, information storage, and communication in living organisms. Scientists emulate these natural assemblies to create modified or synthetic architectures for technological applications [314,315].

Researchers utilize both rational design and laboratory evolution strategies in biomolecular engineering. Rational design involves intuitive, modular, and computer-aided design to modify specific interactions within the assembly [316]. The use of biomolecular templates offers several advantages, including the ability to create complex architectures and to organize materials with nanoscale precision. For example, viral coat proteins, such as those from the tobacco mosaic virus (TMV), can be used as templates for the self-assembly of plasmonic nanoparticles. Petrescu et al. [317] demonstrated the use of tobacco mosaic virus (TMV) coat protein as a template to self-assemble plasmonic nanoparticles into highly symmetrical plasmonic nanorings. This approach exploits the self-assembling properties and chemical addressability of TMV coat protein, using site-directed mutagenesis and bioconjugation strategies, to arrange nanoparticles spatially and control material properties through collective interactions. TMV coat protein is known for its ability to self-assemble into supramolecular nanoparticles, as either protein discs or rods. TMV can also be modified with a small molecular fluorous ponytail at specific sites and self-assemble into spherical nanoparticles through fluorous interaction-induced self-assembly [318]. These resulting macromolecular assemblies can have interesting structural and mechanical properties. Molecular dynamics simulations are valuable for visualizing the stabilization of nuclei at an atomic level during the initial stages of condensation.

Biomolecular templates, including proteins, peptides, and viral particles, direct the assembly of nanomaterials with atomic precision, bridging gaps in conventional lithography. In nanoimprint lithography, flexible biomolecular molds (e.g., elastomer–protein composites) improve conformality on rough substrates, reducing defects [205]. For EUV lithography, peptide-based additives enhance resist sensitivity and etch resistance by modulating acid diffusion and secondary electron yield [319]. Techniques like polymer pen lithography (PPL) exploit biomolecular inks to achieve sub-100 nm resolution with combinatorial patterning capabilities, enabling high-throughput screening of nanostructures for applications in biosensing and nanoelectronics [320]. These templates also support hybrid approaches, integrating self-assembly with optical or electron beam lithography for sub-5 nm node fabrication.

## 10. Conclusions and Future Scope

Lithography technology faces significant challenges in applications like LSI circuit manufacturing and nanophotonics, primarily due to the limits of optical resolution and the desire for miniaturization. As device features shrink below the wavelength of light, traditional photolithography struggles with diffraction limits, necessitating techniques like EUV lithography and multiple patterning to achieve smaller features. Additionally, increasing alignment and overlay precision is crucial as feature sizes shrink, with misalignments leading to yield loss. Advances in metrology, multi-patterning, and machine learning-based alignment systems help address these issues. However, limitations in photoresist materials and mask defects remain a challenge, with the potential for maskless lithography methods to help. Furthermore, the environmental impact of lithography processes, particularly with EUV and CMP, raises concerns, prompting research into greener, more sustainable materials and processes. Overall, overcoming these challenges requires continuous innovation in materials, techniques, and sustainability.

The techniques explored in this review—extreme ultraviolet lithography (EUV), electron beam lithography (EBL), X-ray lithography (XRL), ion beam lithography (IBL), and nanoimprint lithography (NIL)—demonstrate distinct strengths and limitations, each addressing specific challenges in achieving nanoscale precision. EUV lithography emerges as a dominant technique for high-volume manufacturing at advanced nodes, leveraging shorter wavelengths, high-NA optics, and chemically amplified resists to achieve sub-10 nm resolutions. Achieving higher resolution and lower defect rate in EUV and other specialized lithography systems requires precise control of photons and energy transfer. This involves advancing optical systems such as multilayer optics and phase-shifting masks, improving resist materials, optimizing photon source stability and brightness (laser plasma sources), and implementing better process control (quantum control of photons, optical proximity correction, in situ feedback loops). By combining these approaches, it is possible to overcome the limitations of current technologies and continue scaling down to the next generation of semiconductor manufacturing. However, it requires a significant investment in both equipment and operational costs, and while its resolution is ideal, the cost of masks remains a challenge.

Meanwhile, EBL and IBL excel in flexibility and prototyping but face significant throughput constraints. XRL and NIL, with their unique capabilities in high-aspect-ratio patterning and cost-effective replication, offer promising alternatives for niche applications. NIL offers ultra-high resolution and low equipment costs but suffers from process complexity, making it better suited for niche applications or small-scale production. Critical to the success of lithography are innovations in resist materials, mask-making processes, and alignment techniques. Advanced materials such as molybdenum and silicon thin films for EUV masks, hybrid organic–inorganic resists, and metal-assisted chemical etching (MACE) techniques for NIL have pushed the boundaries of what is achievable. Alignment marks and systems now enable real-time corrections, improving overlay precision and throughput. Despite these advancements, challenges such as defect mitigation, mask durability, and stochastic effects persist, particularly as feature sizes approach atomic dimensions. Table 5 shows a comparison of different lithography techniques.

Looking forward, the integration of computational techniques like AI-driven lithographic optimization, the development of alternative lithographic methods such as maskless direct-write systems, and the exploration of emerging materials will be pivotal in overcoming current limitations. Additionally, hybrid approaches combining different lithographic techniques could provide tailored solutions for complex manufacturing needs. In conclusion, while lithography faces significant technical and economic challenges, it remains indispensable in pushing the boundaries of semiconductor technology. As research and development continue to address its limitations, lithography will undoubtedly play a central role in shaping the next generation of electronic devices, from advanced integrated circuits to nanoscale sensors and beyond. Some potential research hotspots and breakthrough points include hybrid lithography systems, advanced resists and materials, fabricating quantum circuits and photonic devices, and developing more sustainable and energy-efficient lithography techniques, such as reducing the power consumption of EUV machines or finding eco-friendly resist materials. This review highlights the ongoing efforts and innovations that ensure lithography remains at the forefront of nanoscale manufacturing, inspiring further exploration and breakthroughs in this critical field.

## Figures and Tables

**Table 1:** Advanced resists for electron beam lithography.

Resist	Resist Type	Developer Solution	Sensitivity (µC/cm^2^)	Contrast	Resolution (nm)	Ref.
40XT	Positive	PEDOT: PSS (0% dilution for 5 s)	8	8 ± 2	95 ± 10	[88]
40XT	Positive	PEDOT: PSS (33% dilution for 40 s)	9	10 ± 0.5	85 ± 5	[88]
ZircSO_x_	Negative	TMAH for 4 min	7.6	2.6	<10	[85]
HafSO_x_	Negative	TMAH for 1.5 min	21	2.5	<10	[85]
ma-N 2400	Negative	ma-D 532	60–160	1.7–2.8	<30	[89]
AR-N 7700	Negative	AR 300-46	8	>5	40–100	[89]
P(HEMA-co-MAAEMA)	Negative	methanol	0.5	1.2	100–200	[89]
Poly (GMA-co-MMA-co-TPSMA)	Negative	7:3 IPA/DI water solution	120		15	[81]

**Table 2:** Types of resists for X-Ray Lithography.

Resist	Resist Type	Incident Dose (mJ/cm^2^)	Resolution (nm)	Wavelength (nm)	Ref.
PMMA	Positive	500	5	0.1–10	[115]
PMMA	Positive	330	35	0.1–10	[115]
CoP (MMA MAA)	Positive	50	<50	1–5	[115]
TIP(MMA-MAA)	Positive	24	<50	1–5	[120]
DQN	Positive	1000	50	0.4–4	[120]
PBOCST	Positive	5	50	0.4–4	[121]
DCIPA	Negative	7.8	500	0.5–5	[121]
Epoxidized polybutadiene	Negative	1.5	1000	0.5–5	[121]

**Table 3:** Types of resists for Ion Beam Lithography.

Resist	Resist Type	Sensitivity (µC/cm^2^)	Contrast	Ref.
PMMA	Positive	2	2.7	[52]
AZ-5206	Positive	1.9	3.3	[52]
OiR-897	Negative	1.4	5	[159]
ARCH	Negative	6.9	30	[159]
AZ-114-PN	Negative	0.12	3.5	[167]
SAL-601	Negative	0.11	3.2	[167]
HSQ	Negative	>10	2–4	[168]
HiPR-6512	Negative	1.0	5.0	[168]
HPR-506	Negative	1.8	3.5	[168]

**Table 4:** Types of materials for Nanoimprint Lithography and their process conditions.

Material	Processing Temperature (°C)	Pressure (MPa)	Process Timing	Ref
PMMA	200	10	3 min	[175]
Polystyrene	160	10	3 min	[175]
Polypropylene	165–225	5	5 min for melting, 30 min for press	[210]
Polyethylene naphthalate	290	2.5	10 min	[211]
Cyclic–olefin copolymer	150	5	5 min	[212]
Polyethylene terephthalate	75–150	2	5 min	[213]
Polycarbonate	160	5	5 min	[214]
Polycarbonate	180	10	10 min	[214]
Polyimide	200	3	2 min	[215]
Epoxy	95	1.2	10 min	[216]
Polyetherimide	285	1	3 min	[217]
Fluorinated Ethylene Propylene	270	0.18	5 min	[218]
Ethylene tetrafluoroethylene	250	1.38	10 s	[219]
Cellulose Acetate	115	13.6	0.2 m/min	[220]

**Table 5:** Comparison of different lithography techniques.

	EUV Lithography	Electron Beam Lithography	X-Ray Lithography	Ion Beam Lithography	Nanoimprint Lithography
Wavelength	~13.5 nm	~1–10 nm	~1–10 nm (soft X-rays)	~10–100 nm	N/A
Throughput	High (production level)	Slow	Moderate	Slow	Very fast
Resolution	~5–7 nm	sub 10 nm	~10 nm	~10 nm	~10–20 nm
Cost	High	High	High	High	Moderate
Masks	Required	No masks	Required	FIB: No masks PBW: No masks IPL: Required	No masks
Advantages	High resolution, well-suited for mass production	High resolution, not for mass production	High resolution	High precision	High throughput
Disadvantages	Complex and expensive infrastructure, limited material availability	Slow speed, expensive	Complex, limited material interactions	Slow, expensive, resolution limited by beam interactions	Limited to specific materials, requires molds

## Data Availability

The authors will provide available data or assist in finding them in the databases.
